# Cystathionine γ-lyase inhibits mitochondrial oxidative stress by releasing H_2_S nearby through the AKT/NRF2 signaling pathway 

**DOI:** 10.3389/fphar.2024.1374720

**Published:** 2024-07-23

**Authors:** Tingting Xiao, Shun Chen, Ge Yan, Junmin Zheng, Qingzhu Qiu, Shujia Lin, Yanfang Zong, Haishuang Chang, Alex Chia Yu Chang, Ying Wu, Cuilan Hou

**Affiliations:** ^1^ Department of Cardiology, Shanghai Children’s Hospital, School of Medicine, Shanghai Jiao Tong University, Pediatric Allergy and Immune Disease Laboratory, Kawasaki Disease Research Center, NHC Key Laboratory of Medical Embryogenesis and Developmental Molecular Biology, Shanghai Key Laboratory of Embryo and Reproduction Engineering, Shanghai, China; ^2^ Shanghai Institute Precision Medicine, Ninth People’s Hospital, School of Medicine, Shanghai Jiao Tong University Shanghai, Shanghai, China; ^3^ Department of Pathology, Shanghai Children’s Hospital, School of Medicine, Shanghai Jiao Tong University, Shanghai, China

**Keywords:** hydrogen sulfide, accelerated aging, mitochondria, PI3K-AKT pathway, cystathionine γ-lyase

## Abstract

Cystathionine γ-lyase (CSE) is a major enzyme that produces hydrogen sulfide (H_2_S). Herein, we report how CSE plays a previously unknown role in regulating the antioxidant effects of the mitochondria in human umbilical vein endothelial cells by releasing H_2_S nearby under stress conditions. We found that H_2_S partially promoted angiogenesis in the endothelial cells through the AKT/nuclear factor erythroid 2-related factor 2 (AKT/NRF2) signaling pathway. H_2_S improved mitochondrial function by altering the expressions of the mitofusin2 and dynamin-1-like mitochondrial fission proteins to inhibit oxidative stress and enhance NRF2 nuclear translocation. CSE is located only in the cytoplasm and not in the mitochondria, but it is transported to the vicinity of the mitochondria to produce H_2_S, which plays an antioxidant role in human umbilical vein endothelial cells under stress. The CSE mutant (with mutated CSE activity center: CSE^D187A^) partially decreased the effects on promoting angiogenesis, resisting oxidative stress, and entering the mitochondria. These results show that CSE translocation is a unique mechanism that promotes H_2_S production inside the mitochondria under stress stimulation. Therefore, the CSE mutant site (CSE^D187A^) may be a potential target for drug therapy.

## 1 Introduction

Hydrogen sulfide (H_2_S) is a physiological signaling molecule that has been shown to have powerful protective effects in multiorgan systems (Szabo, 2007). H_2_S is mainly produced in the body by three enzymes: cystathionine γ-lyase (CSE), cystathionine beta-synthase, and 3-mercatopyruvate sulfur transferase. CSE regulates the cysteine biosynthesis pathway and plays crucial roles in blood pressure, cellular metabolism, and angiogenesis (Cai et al., 2007; Wang, 2012). Fu et al. (2012) reported that CSE can translocate into the mitochondria under stressed conditions, while Ellwood et al. (2021) showed that mitochondrial H_2_S supplementation can improve C*aenorhabditis elegans* health. Lohakul et al. (2021) showed that targeting H_2_S delivery to the mitochondria is a novel method of preventing and treating the photoaging of skin. Therefore, CSE translocation may play a crucial role in living organisms.

In this study, we found that administering and activating endogenous H_2_S played a protective role in the D-galactose (Dgal)-induced accelerated aging of human umbilical vein endothelial cells (HUVECs). Endothelial protection is associated with upregulation of the metabolic pathways and improvement of mitochondrial functions (including ATP production, basal and maximal respiration), while partially blocking phosphatidylinositol-3-kinase (PI3K)/AKT and nuclear factor erythroid 2-related factor 2 (NRF2) inhibitors. Under Dgal-induced stress conditions, once the activity center of CSE mutated (CSE^D187A^), its translocation and protective effects disappeared. The findings of this study suggest that H_2_S has cytoprotective effects on the accelerating aging of endothelial cells and may be clinically important.

## 2 Materials and methods

### 2.1 Cell cultures and cell proliferation assay

Chen et al. (2019) showed that 10 g/L (almost 50 mM) of Dgal-induced HUVECs can mimic accelerated senescence, and our previous study showed that 50 mM of Dgal-pretreated HUVECs can also mimic accelerated senescence (Hou et al., 2016). Therefore, we selected 50 mM of Dgal to establish a cell senescence model. The Cell Counting Kit-8 (CCK-8, E606335-0500, BBI Life Sciences) was used to detect cell proliferation; cells were cultured in a 96-well plate (Corning, United States) and exposed to Dgal (50 mM) for 46 h, after which the culture medium was removed. Then, 10 μL of the CCK-8 detection solution was added with 100 μL of endothelial cell medium (ECM). The background control was composed of 10 μL of CCK-8 and 100 μL of ECM without cells. After incubating the samples in a cell incubator at 37°C, the absorbance was detected at 450 nm using a microplate reader (Thermo Fisher Scientific, United States). The experiments were repeated at least thrice.

### 2.2 Measurement of H_2_S levels

Plasma levels of H_2_S (C57Bl/6 J, CSE^−/+^, and CSE^−/−^ mice) and HUVECs were detected as described previously (Shen et al., 2011). All experiments were performed in accordance with the guidelines of the Ethics Committee of Experimental Research at Shanghai Children’s Hospital, School of Medicine, Shanghai Jiao Tong University. First, approximately 30 μL of blood and/or HUVEC lysates (0.5% ammonia was used to lyse the HUVECs) was incubated with 10 µL of ammonia (0.1%) and 80 μL of monobromobimane (MBB) for 40 min at room temperature. The reaction was terminated by adding 20% formic acid and analyzed using gas chromatography mass spectrometry (GC-MS). The experiments were performed in a double-blind manner for detecting the H_2_S levels.

### 2.3 RNA extraction and gene expression analysis

Total RNA of the HUVECs was extracted using RNAiso (TaKaRa, Beijing, China), and its integrity was evaluated using a Bio-analyzer 2100 system (Agilent Technology, CA, United States). A sequencing library was then constructed; the library was sequenced on an Illumina HiSeq 3500 platform to generate 150-bps-long paired-end reads. The reading counts for each sample were analyzed using HTSeq v6.0. The reads per kilo base million mapped reads (RPKM) were computed to estimate the gene expression levels. The gene ontology (GO) and Kyoto Encyclopedia of Genes and Genomes (KEGG) enrichment databases (http://www.genome.jp/kegg/) were used. Benjamini-corrected *p* < 0.05 was used as the cutoff value for significantly enriched biological processes, and the raw and clean data were obtained after filtering for quality control.

### 2.4 Immunofluorescence analyses

The HUVECs were fixed in 4% paraformaldehyde for 15 min. After blocking, the cells were stained with NRF2 (Proteintech, United States), CD31, γ-H2AX, proliferating cell nuclear antigen (PCNA) (Cell Signaling Technology, United States), or cystathionine γ-lyase (CTH) antibody (Abcam, United States). A secondary antibody (Invitrogen, United States) and DAPI were used for staining, and the cells were washed with a TBST buffer. Images were then obtained using a laser confocal microscope (Zeiss LSM710, Germany). Ten fields were randomly selected for each group, and all histological examinations were performed in a blinded manner.

### 2.5 Cell migration assay

The HUVECs were starved for 12 h with hydroxyurea (5 mM; Sigma-Aldrich, Germany) to inhibit cell proliferation before pretreatment with Dgal (25 mM) for 24 h. The monolayer was transfected with CSE green-fluorescent protein (GFP)/CSE-GFP-mut (CSE^D187A^) or non-specific negative controls (GFP) and scraped with a 200-μL pipette tip to generate scratch wounds. The monolayer was then rinsed twice with ECM containing 1% fetal bovine serum. After a series of treatments including DL-propargylglycine (PPG, 2 mM), NaHS (50 μM), Nac (5 mM), vehicle (0.5% dimethyl sulfoxide; DMSO), Ly294002 (1 μM), and Ml385 (2 μM) for 24 h, the cells were photographed immediately and again after 24 h with a PE Operetta system (PerkinElmer, United States). All migration assays were performed in a blinded fashion and analyzed using ImageJ software.

The tube-like network formed on a Matrigel (BD Bioscience, United States) basement membrane matrix was added to a 24-well culture plate and incubated at 37°C until gelation (Wara et al., 2011). In brief, the HUVECs were transfected with CSE-GFP/CSE-GFP-mut or non-specific negative controls (GFP), cultured for 48 h, pretreated as described above for wound healing, and plated onto Matrigel at a rate of 30,000 cells/well. The branching points and tube lengths were quantitatively determined from five random microscopic fields.

### 2.6 *In vitro* cell apoptosis detection assay

Annexin V fluorescein isothiocyanate (FITC)/propidium iodide (PI) kits (Sigma-Aldrich, Germany) were used to detect apoptosis in the living cells (Hou et al., 2017). For the exogenous H_2_S assay, the cells in the experimental group were pretreated with Dgal (25 mM) for 24 h and then treated with PPG, NaHS, or vehicle (0.5% DMSO) for another 24 h. The control group was treated with the vehicle (0.5% DMSO) for 48 h. For the endogenous H_2_S assay, the cells were pretreated with Dgal (25 mM) and transfected with CSE-GFP or the non-specific negative control (GFP) for 24 h, before being treated with Ly294002, Ml385, or the vehicle (0.5% DMSO) for another 24 h. After treatment, the cells were collected, stained with Annexin V-FITC/PI, and analyzed by flow cytometry (FCM) using an Annexin V-FITC cell apoptosis detection kit according to manufacturer instructions.

### 2.7 Caspase activities in the HUVECs

Caspase-3, -8, and -9 protease assay kits (Thermo Fisher Scientific, United States) were used according to manufacturer instructions. The assay loading solutions for each substrate (caspase 3, 8, and 9) were prepared, mixed well, and incubated at room temperature for 30–60 min. The HUVECs were then seeded on a 96-well plate at a density of 2 × 10^4^ cells/90 µL. The cells were pretreated with Dgal (25 mM) and transfected with CSE-GFP or a non-specific negative control (GFP) for 24 h, followed by treatment with Ly294002, Ml385, or the vehicle (0.5% DMSO) for another 24 h. The fluorescence intensity was monitored using a fluorescence microplate reader at the following excitation/emission wavelengths: Caspase 3 = 535/620 nm (red); Caspase 8 = 490/525 nm (green); Caspase 9 = 370/450 nm (blue).

### 2.8 Transmission electron microscopy

TEM (FEI Talos L 120C, Thermo Fisher Scientific, United States) was performed for morphological analysis at the Shanghai Institute of Precision Medicine, Ninth People’s Hospital, School of Medicine, Shanghai Jiao Tong University. For morphological TEM, acute isolated cardiomyocytes were fixed overnight with ice-cold 2.5% glutaraldehyde at 4°C. The ultrathin sections were then stained with uranyl acetate and lead citrate. Next, sections of 90–100 nm thickness were mounted on a 200-mesh copper grid and imprinted using an FEI Tecnai G2 Spirit transmission electron microscope (Thermo Fisher, MA, United States). Six to eight visual fields were randomly selected to analyze the number of mitochondria using ImageJ software. All the analyses were performed with observer blinding.

### 2.9 Seahorse assay

Mitochondrial fuel usage in the living cells was detected using the Seahorse XF cell Mito Stress Test Kit (Agilent Technologies, Santa Clara, CA, United States). The cells were transferred to a Seahorse XFp system and analyzed using Wave software (Agilent Technologies, Santa Clara, CA, United States). On the day prior to the experiments, the HUVECs (5000/well) were seeded in Seahorse XFp96 cell culture miniplates for 48 h, and a sensor cartridge was hydrated in the Seahorse XF calibrant overnight at 37°C in a non-CO_2_ incubator. On the day of the experiments, the XF cell mito stress test medium (XF base medium, 1 mM pyruvate, 2 mM glutamine, and 10 mM glucose warmed to 37°C and adjusted to pH 7.4 with 0.1 M NaOH) was prepared. The cells were transferred and incubated with the appropriate assay medium for 1 h in a non-CO_2_ incubator at 37°C. Pouches containing the compounds oligomycin, trifluoromethoxy carbonyl cyanide phenylhydrazone (FCCP), and rotenone/antimycin were incubated at room temperature for 15 min. Lastly, the compounds were resuspended with the prepared assay medium and diluted to obtain the following final concentrations: 10 μM oligomycin, 10 μM FCCP, and 5 μM rotenone/antimycin for the mito stress test.

### 2.10 Fluorescent detection of reactive oxygen species and MitoSox

Intracellular ROS levels were measured by dihydroethidium (DHE) staining (Sigma-Aldrich, Germany) and CellROX. For the DHE staining, the HUVECs were incubated with 1 μM DHE (dilution with phosphate-buffered saline (PBS)) for 30 min and observed under a laser confocal microscope (Zeiss LSM710, Germany) at excitation/emission wavelengths of 488/610 nm. For the CellROX assay, the HUVECs were incubated with 5 μM CellROX for 30 min and read on a FlexStation 3 multimode microplate reader (VWR Corporation, Radnor, United States). For the mitochondrial superoxide assay, the HUVECs were incubated with 5 μM MitoSox reagent (Invitrogen, United States) working solution for 30 min and observed under a laser confocal microscope at excitation/emission wavelengths of approximately 510/580 nm before being read on the FlexStation 3 microplate reader.

### 2.11 Immunoelectron microscopy

The HUVECs were seeded onto 3 mm sapphire discs; the sapphire disc was then placed with the cells facing up on a flat aluminum planchette, and the inner space of the other aluminum planchette (25 µm) was used as a cover. The space between the two aluminum planchettes was filled with 1-hexadecane. The samples were immediately frozen using an EM ICE high-pressure freezing machine (Leica, Germany) and rapidly stored in liquid nitrogen. The samples were first incubated in acetone containing 0.2% uranyl acetate (UA; −90°C, 48 h) and then at −50°C for 4 h before repeating the process. The samples were next incubated in acetone containing 0.2% UA (12 h) and then at −30°C for 4 h. Then, the samples were rinsed three times with pure acetone (15 min each) and gradually infiltrated in HM20 resin of grades 25%, 50%, 75%, and pure resin (1 h each) at −30°C. The samples were embedded in gelatin capsules after overnight infiltration in the pure resin and polymerized under UV light at −30°C (48 h) and 25°C (12 h). After polymerization, the samples were trimmed and ultrathin sectioned using a microtome (Leica UC7, Germany). Serial thin sections (100 nm thick) were collected on formvar-coated nickel grids and incubated in 0.01 M PBS (including of 1% bovine serum albumin, 0.05% Triton X-100, and 0.05% Tween 20) for 5 min. The sections were next incubated in the primary antibody CTH overnight at 4°C and then in the secondary antibody (goat anti-rabbit conjugated with 10 nm gold) for 2 h at room temperature. The sections were finally washed with PBS, dried at room temperature, and examined by TEM (FEI Talos L 120C, Thermo Fisher Scientific, United States).

### 2.12 Cell lysate and immunoblotting

The cytoplasmic and nuclear proteins were collected using a nuclear and cytoplasmic protein extraction kit (Beyotime Biotechnology, Nanjing, China) and quantified using a BCA kit (Shen Neng Bo Cai Corp., Shanghai, China). Whole proteins were lysed with a cell lysate buffer (containing radioimmunoprecipitation assay, protease inhibitors, and phosphatase inhibitors) and quantified. The proteins were resolved using sodium dodecyl-sulfate-polyacrylamide gel electrophoresis and transferred onto polyvinylidene fluoride membranes (Millipore, Bedford, MA, United States). The membranes were incubated with primary antibodies specific to NRF2, lamin B1, and glyceraldehyde-3-phosphate dehydrogenase (GAPDH, Cell Signaling Technology, United States) at a dilution of 1:1,000 in a blocking buffer. The membranes were then incubated with secondary horseradish-peroxidase-conjugated antibodies (Cell Signaling Technology, United States) at a dilution of 1:2,000. After washing, the membranes were visualized using a chemiluminescent substrate (ECL) kit. The immunological band densities were analyzed via a scanning densitometer (GS-800, Bio-Rad Laboratories, Hercules, CA, United States) coupled with the Bio-Rad personal computer analysis software.

### 2.13 Statistical analysis

Tandem mass spectra were processed using PEAKS Studio version X (Bioinformatics Solutions Inc, Canada). The peptides were filtered with 1% FDR; the differently expressed proteins were filtered if they contained at least one unique peptide with significance over 13 (*p* < 0.05) with a fold change over 1.3. The results were expressed as mean ± standard error of the mean (SEM). Statistical analyses were performed using SPSS software, version 21.0 (SPSS, Inc., United States), and comparisons among the groups were performed by one-way ANOVA. The paired data were evaluated by two-tailed Student’s t-test. The data were considered to be statistically significant at *p* < 0.05.

## 3 Results

### 3.1 Decreased H_2_S levels are common in aging

**FIGURE 1:**
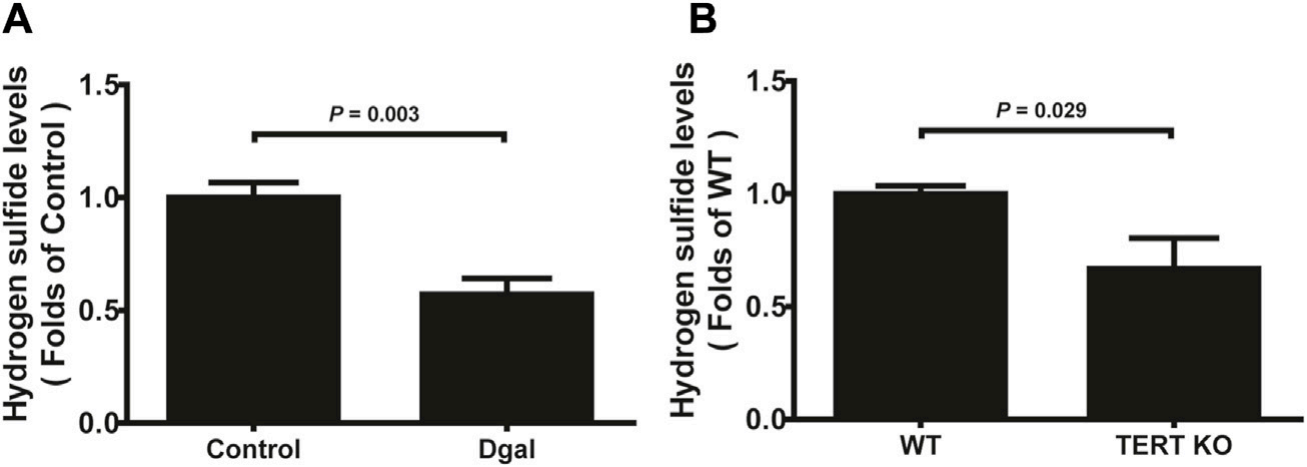
H_2_S levels in D-galactose (Dgal)-induced accelerated aging of HUVECs. **(A)** H_2_S levels in the HUVECs (n = 6). **(B)** H_2_S levels in the TECR-knockout mouse plasma (n = 5). The values are expressed as means ± SEM, with *p* < 0.05 considered as significant.

**FIGURE 2:**
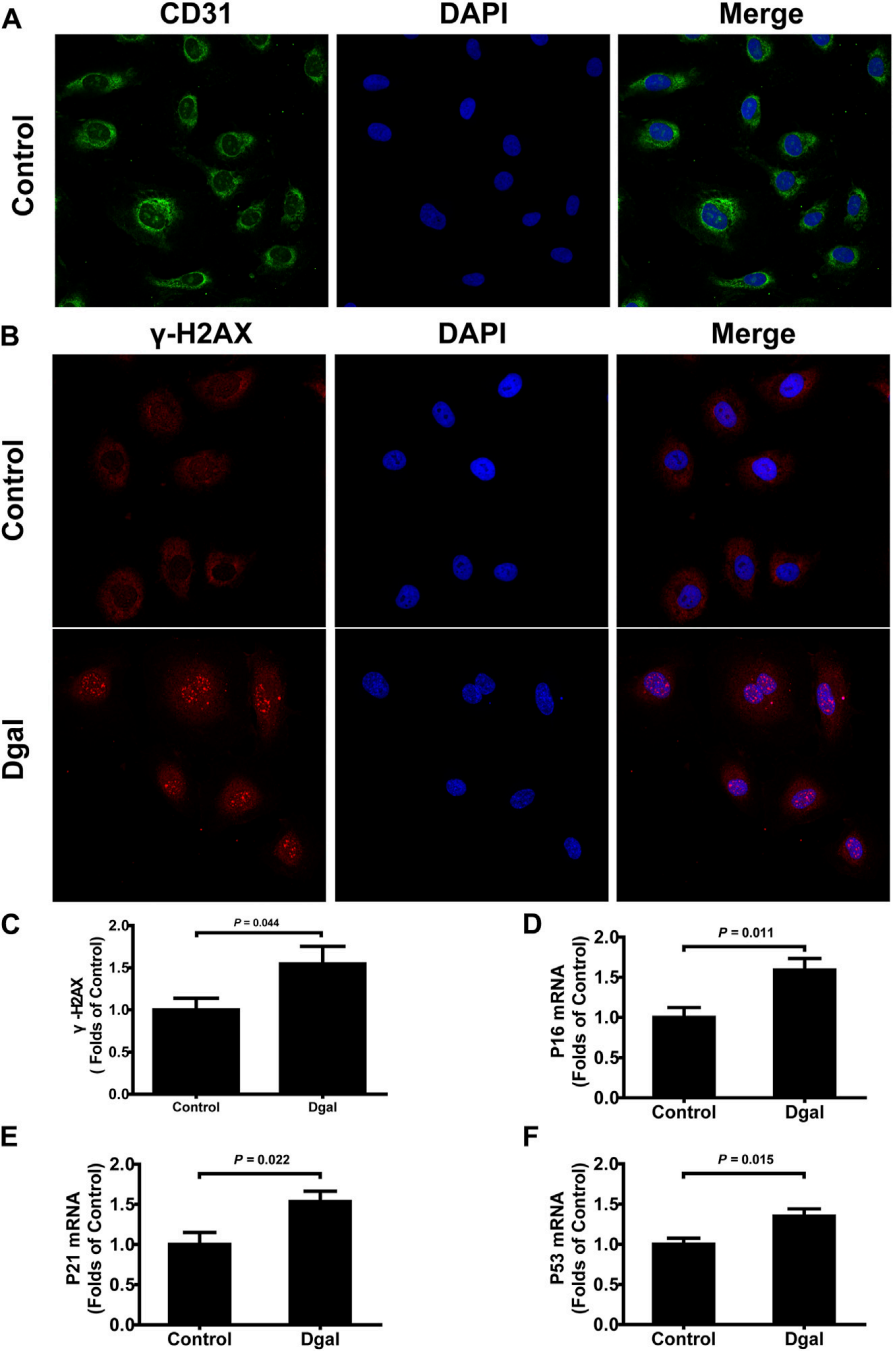
Characterization of Dgal-induced accelerated senescence of the HUVECs. **(A)** Representative immunofluorescence images of CD31 in the HUVECs (n = 3). **(B, C)** Representative immunofluorescence images and quantification of γ-H2AX in the HUVECs (n = 7). **(D–F)** Quantifications of the mRNA levels of P16, P21, and P53 in the HUVECs (n = 6). The values are expressed as means ± SEM, with *p* < 0.05 considered as significant.

Our previous study showed that H_2_S levels decreased in the Dgal-induced accelerated mouse heart, liver, and kidney tissues (Wu et al., 2017) as well as decreased in the naturally aging mouse kidneys and plasma (Hou et al., 2016). In the present study, we found that the H_2_S levels decreased in Dgal-induced accelerated senescence of endothelial cells *in vitro* (Figure 1A) and in TERT-knockout mouse (TERT-knockout model, telomerase-deficient mouse) plasma (Figure 1B). Therefore, the decrease in H_2_S level may be a common phenomenon during aging. We then tested whether a Dgal-induced accelerated aging model could be constructed successfully. The HUVECs were positively stained with endothelium marker CD31 and DNA damage marker γ-H2AX after incubation with Dgal (25 mM) for 48 h (Figures 2A–C). The HUVECs also highly expressed aging markers, such as p16, p21, and p53, indicating that the accelerated aging cell model was built (Xu et al., 2018) (Figures 2D–F).

### 3.2 CSE upregulates the endothelium metabolism pathway

**FIGURE 3:**
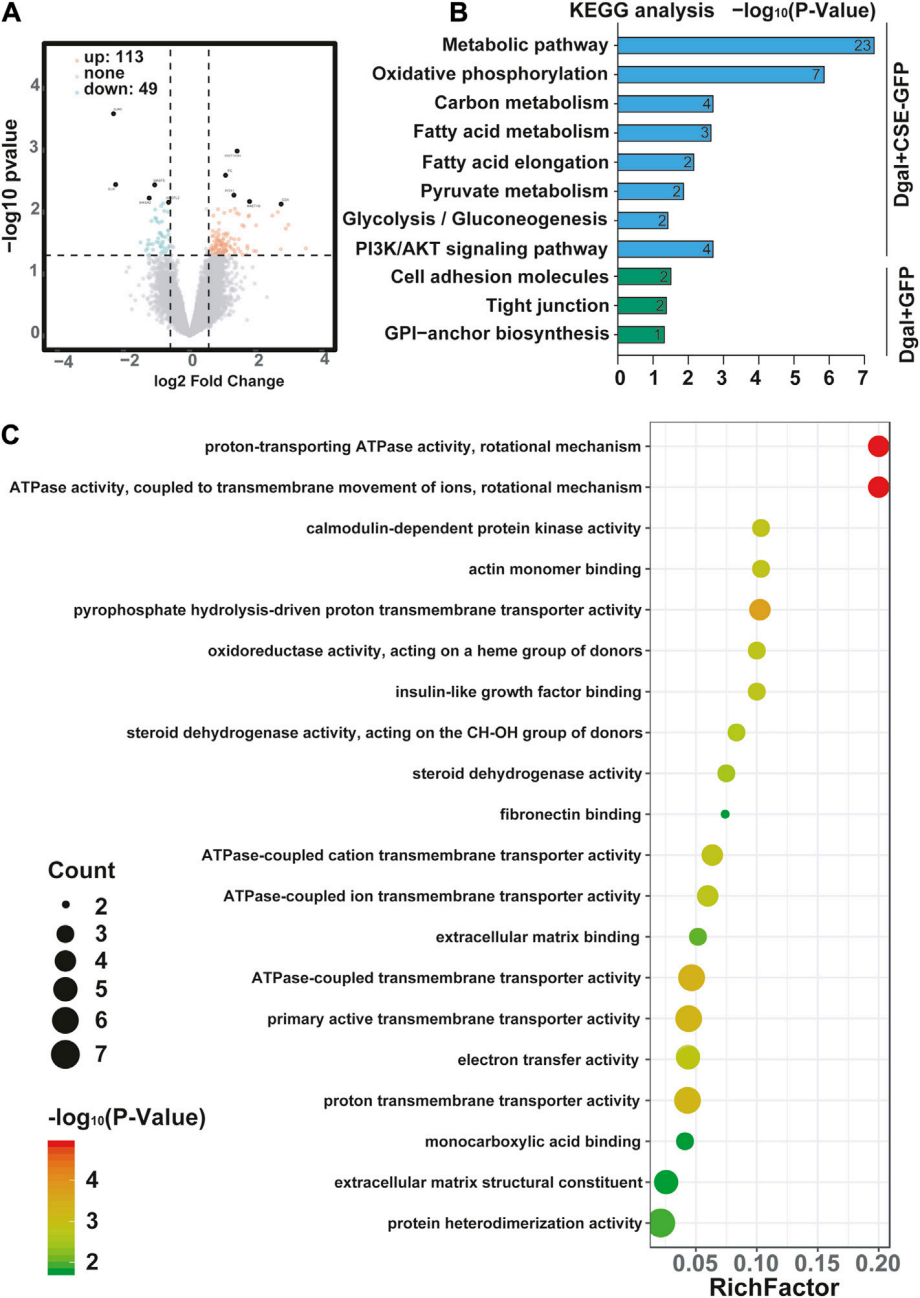
Visualization of the differentially regulated mRNAs in the Dgal-induced accelerated aging HUVECs upon CSE overexpression. **(A)** Representative volcano images of the differentially regulated mRNAs in the Dgal + GFP and Dgal + CSE-GFP groups, highlighted for those achieving *p* < 0.05 and |fold-change| > 1.3. **(B)** KEGG enrichment analyses of the differentially expressed mRNAs. **(C)** GO enrichment analyses of the differentially expressed mRNAs (n = 3).

We profiled the Dgal-induced accelerated senescence of the HUVEC transcriptome using RNA-seq to assess the effects of CSE. The CSE mRNA levels increased significantly after CSE overexpression (Supplementary Figure S1A). We identified 162 differentially expressed genes, of which 113 (69.8%) were upregulated and 49 (30.2%) were downregulated after CSE overexpression (Supplementary Table S1 and Figure 3A). The H_2_S levels increased significantly in the accelerated senescent HUVECs after CSE overexpression (Supplementary Figure S1B). KEGG enrichment analysis was performed to identify the potentially affected pathways; herein, we observed that the metabolic pathway, oxidative phosphorylation, pyruvate metabolism, and PI3K/AKT signaling pathway were enriched after infection of the HUVECs with the CSE adenovirus (Figure 3B) (*p* < 0.05). Similarly, GO enrichment analysis revealed that these differentially expressed genes were involved in proton-transporting ATPase activity, rotational mechanisms, ATPase activity, couples in transmembrane movement of ions, and ATPase-coupled ion transmembrane transporter activity (Figure 3C) (*p* < 0.05). Collectively, these data suggest that CSE plays a key role in endothelial metabolism.

### 3.3 H_2_S promotes endothelial cell proliferation, migration, and tube formation

**FIGURE 4:**
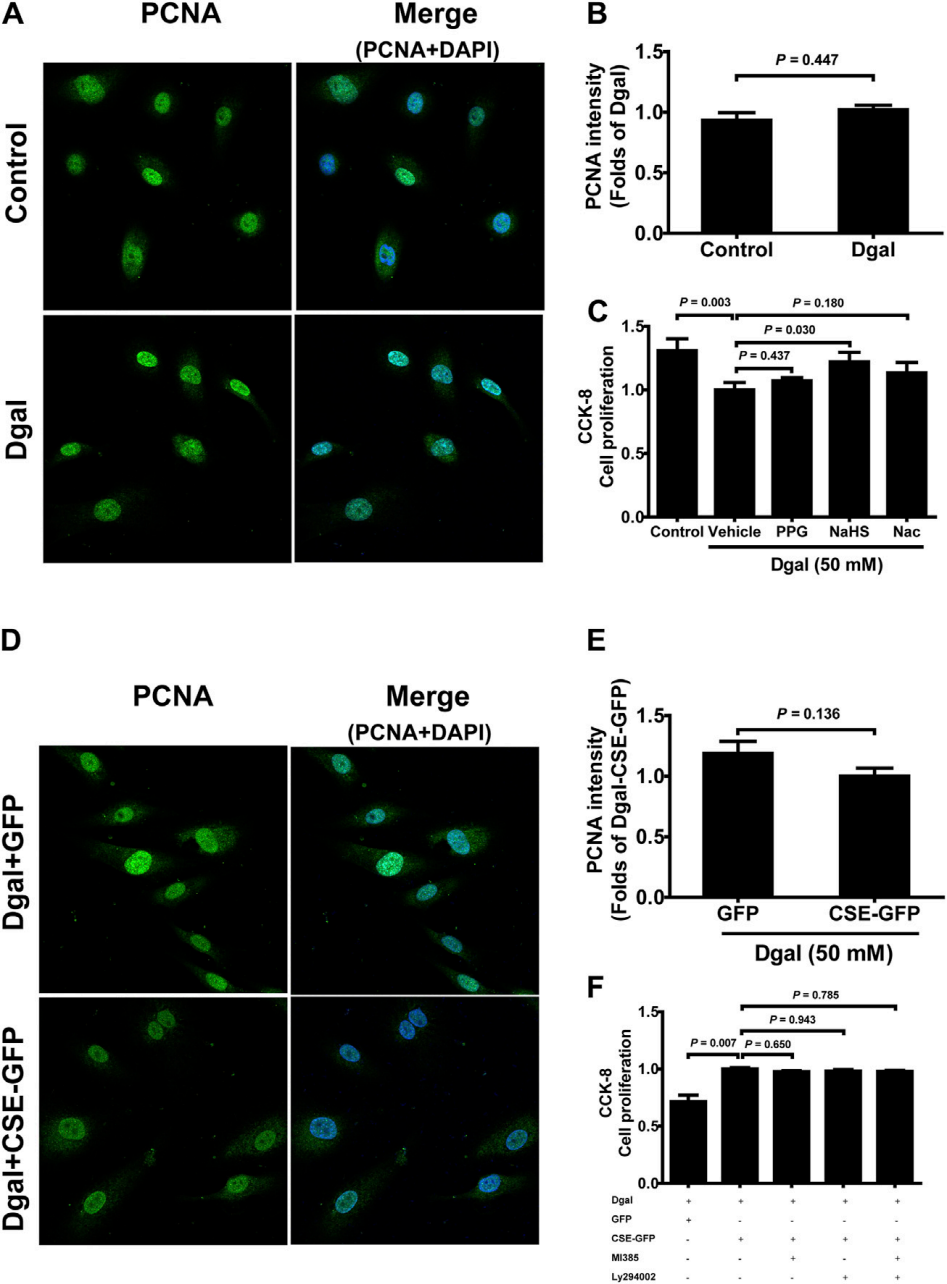
Influence of NaHS treatment on HUVEC proliferation ability. **(A, B)** Representative immunofluorescence images and statistical analysis of PCNA intensity between the control and Dgal-induced HUVECs (n = 6). **(C)** Statistical analysis of the effects of exogenous H_2_S on cell proliferation determined by the CCK-8 assay (n = 8). **(D, E)** Representative immunofluorescence images and quantification of PCNA intensity in the Dgal-induced HUVECs upon CSE overexpression (n = 6). **(F)** Statistical analysis of the effects of endogenous H_2_S on cell proliferation determined by the CCK-8 assay (n = 9). The values are expressed as means ± SEM, with *p* < 0.05 considered as significant.

**FIGURE 5:**
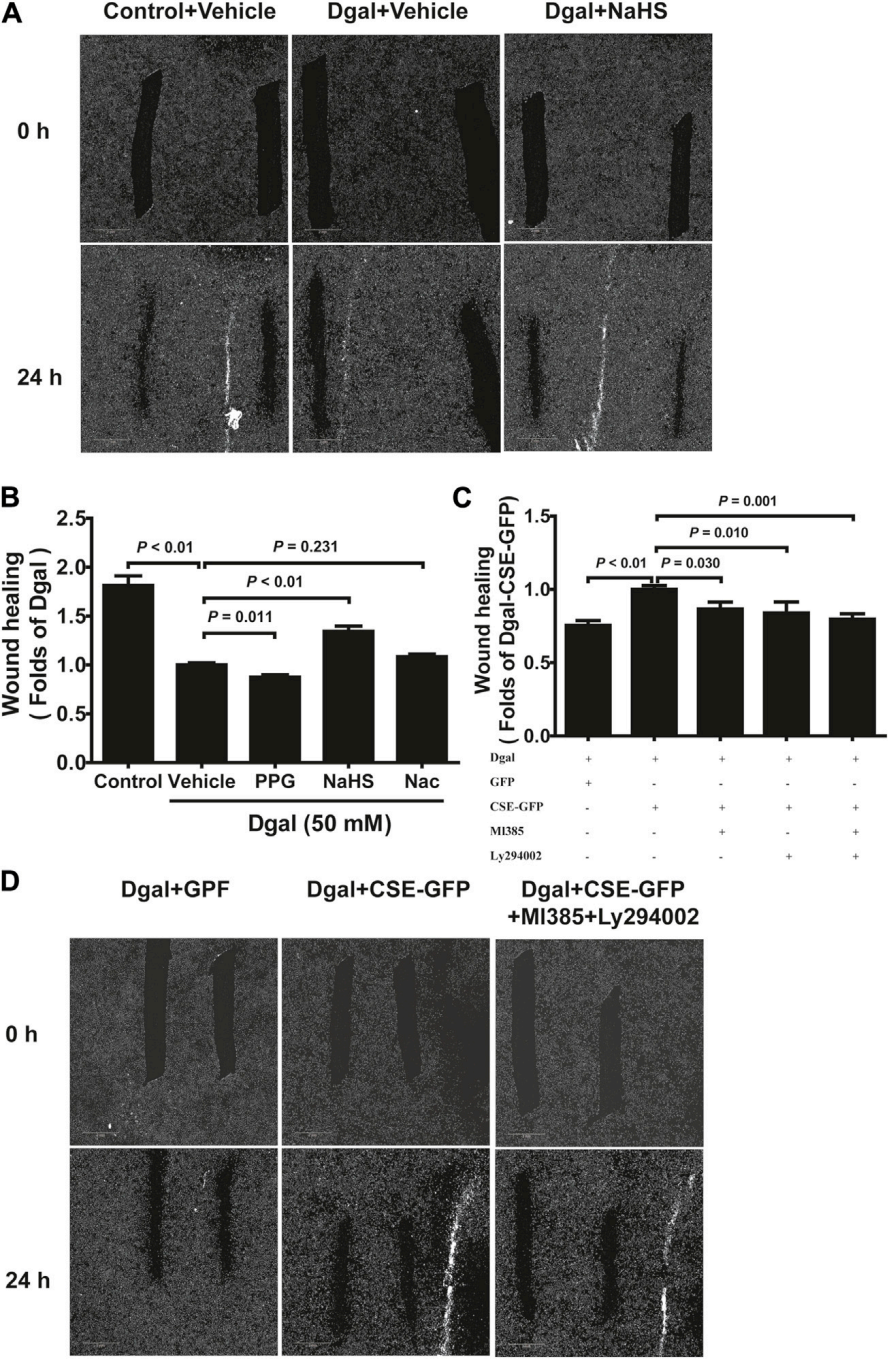
H_2_S-promoted endothelial cell migration is mitigated by PI3K/AKT signaling. **(A, B)** Representative scratch wound healing micrographs of exogenous H_2_S treatment (0 h and 24 h) and statistical charts (n = 10). Scale bar = 2 mm. **(C, D)** Statistical charts and representative scratch wound healing micrographs of endogenous H_2_S treatment (0 h and 24 h) (n = 8). Scale bar = 2 mm. The values are expressed as means ± SEM, with *p* < 0.05 considered as significant.

**FIGURE 6:**
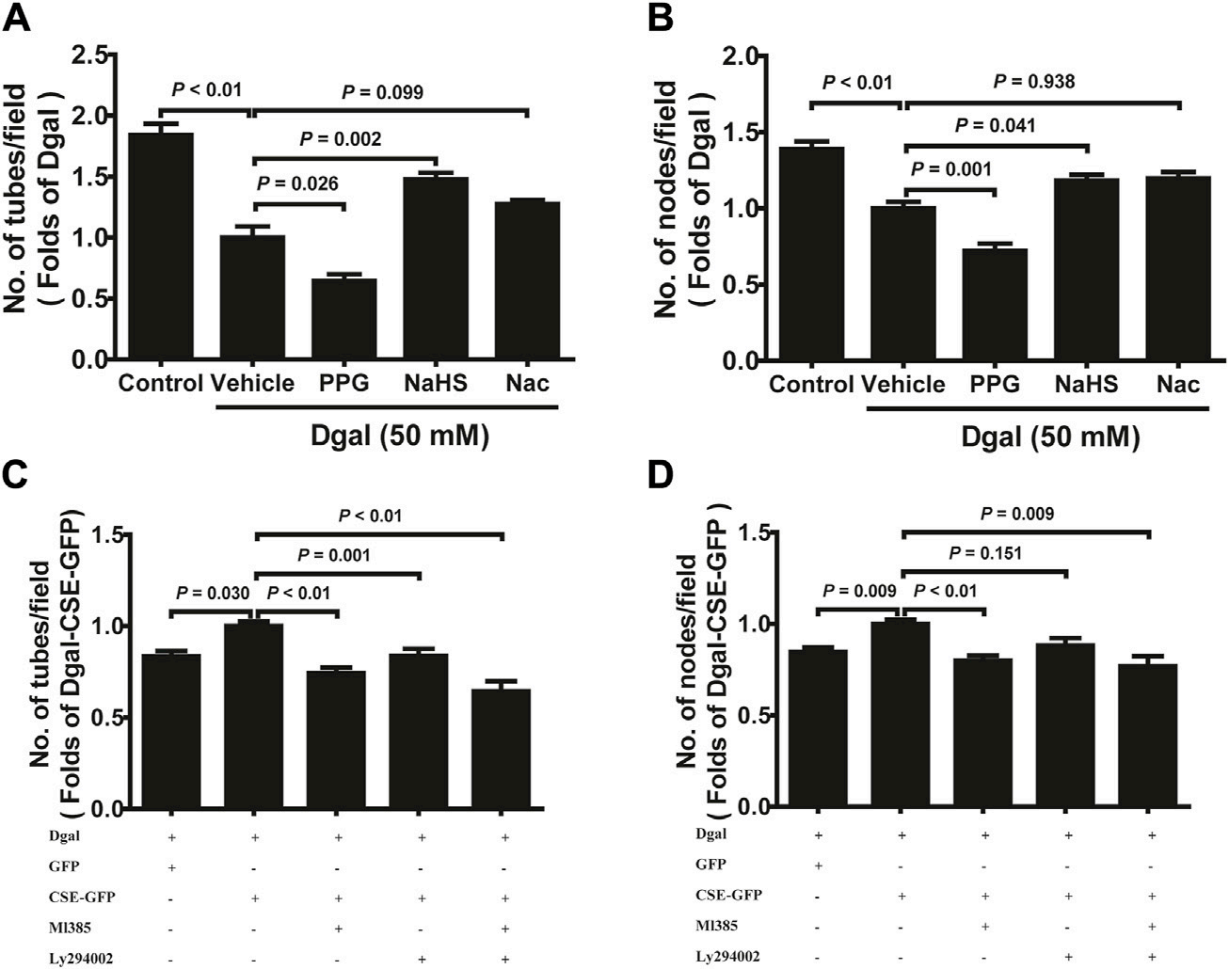
H_2_S promoted endothelial cell tube formation. **(A, B)** Statistical charts of the tubes and branching point number for treatment with PPG, NaHS, or Nac (n = 15). **(C, D)** Statistical charts of the tubes and branching point number for treatment with CSE overexpression with or without Ml385 or Ly294002 (n = 12). The values are expressed as means ± SEM, with *p* < 0.05 considered as significant.

We selected 10, 50, and 100 μM of NaHS as the exogenous H_2_S donors and noted that 50 μM NaHS had better antiaging effects on Dgal-induced accelerated senescent HUVECs (Topf et al., 2018). Thus, we directly selected 50 μM NaHS. We then tested the effects of H_2_S on the endothelial cells and whether they were regulated by the PI3K/AKT signaling pathway. PCNA staining was not significantly different between the control and Dgal groups (Figures 4A,B), while the H_2_S-stimulated endothelial cell viability was dependent on PI3K/AKT and NRF2 signaling via the CCK-8 assay (Figure 4C; Supplementary Figure S1C). Moreover, CSE overexpression did not alter PCNA staining (Figures 4D,E) but rather promoted endothelial cell viability, in addition to not being dependent on PI3K/AKT and NRF2 signaling (Figure 4F). The exogenous H_2_S also promoted angiogenesis in the accelerated-aging endothelial cells depending on PI3K/AKT and NRF2, which abated upon treatment with the CSE inhibitor, PPG, in the scratch wound healing assay (Figures 5A,B; Supplementary Figure S1D). CSE overexpression accelerated the aging endothelial cell migration, which was blocked by the PI3K/AKT and NRF2 inhibitors (Figures 5C,D). The angiogenesis promotion was also confirmed by the tube formation assay; H_2_S promoted angiogenesis in the accelerated senescent endothelial cells, which abated upon treatment with the CSE inhibitor, PPG (Figures 6A,B). CSE overexpression promoted accelerated senescent endothelial cell tube formation and was blocked with an NRF2 inhibitor but not with the PI3K/AKT inhibitor (Figures 6C,D).

### 3.4 H_2_S alleviates Dgal-induced apoptosis and caspase activity

**FIGURE 7:**
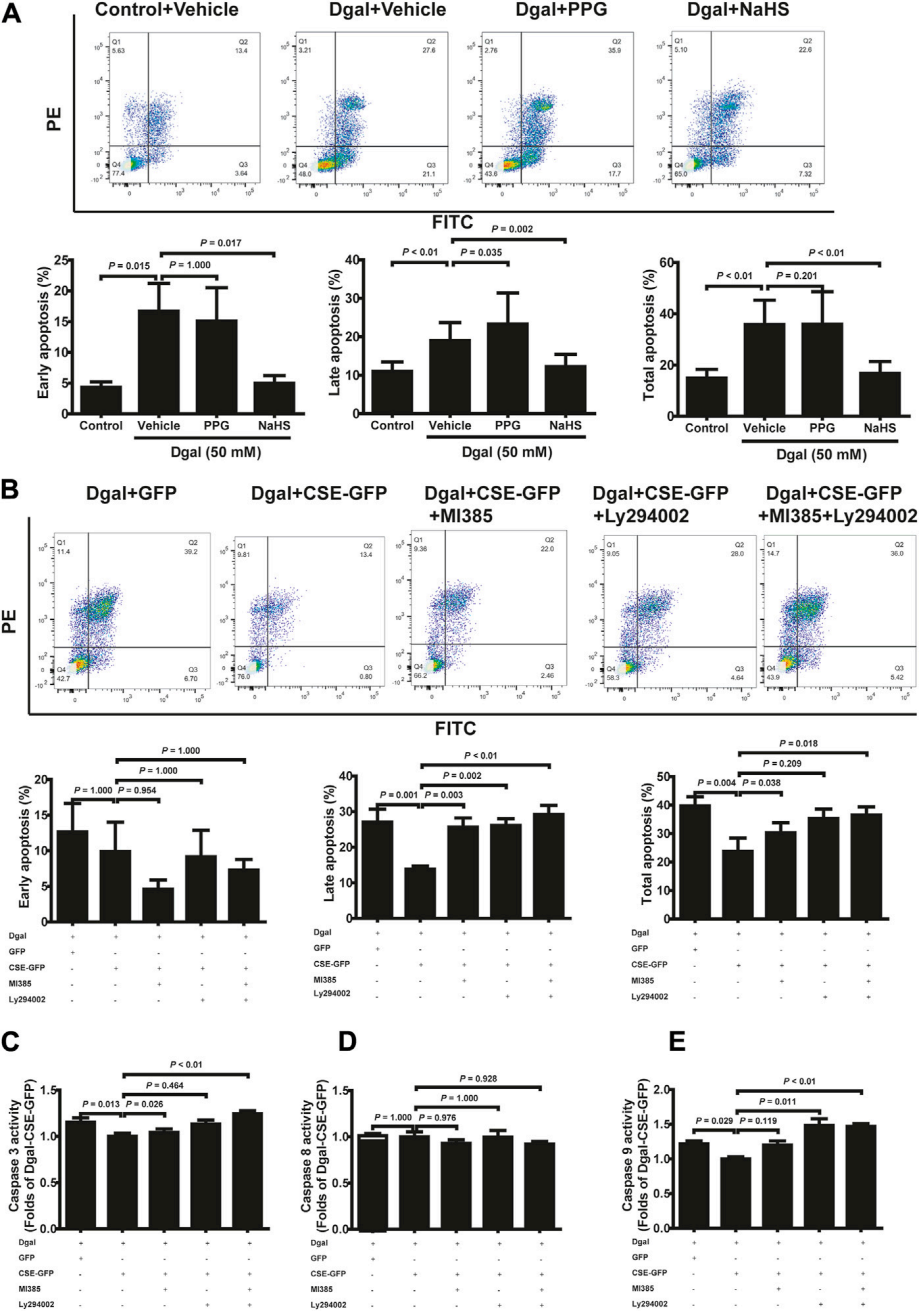
H_2_S decreased Dgal-induced endothelial cell apoptosis, and Caspase activities were mitigated by PI3K/AKT signaling. **(A)** Representative flow cytometry images and statistical charts for exogenous H_2_S treatment (n = 7). **(B)** Representative flow cytometry images and statistical charts for endogenous H_2_S treatment (n = 6). **(C, D, E)** Statistical charts of caspase 3, 8, and 9 activities for endogenous H_2_S treatments (n = 6). The values are expressed as means ± SEM, with *p* < 0.05 considered as significant.

To validate the protective effects of the CSE-PI3K/AKT signaling axis, we measured the senescent endothelial cell apoptosis. We observed the antiapoptotic (including early, late, and total apoptosis) effects of H_2_S in the HUVECs, which abated after PPG treatment (only affected late apoptosis but not early and total apoptosis), using FCM (Figure 7A). CSE overexpression decreased the apoptosis of accelerated senescent endothelial cells (including late and total apoptosis); late apoptosis was blocked by the PI3K/AKT and NRF2 inhibitors, whereas total apoptosis was blocked by the NRF2 inhibitor (Figure 7B). These effects were confirmed using the caspase family: the activities of caspase 3 and 9 decreased upon CSE overexpression; caspase 3 was blocked with the NRF2 inhibitor, caspase 9 was blocked with a PI3K/AKT inhibitor, and caspase 8 remained unaffected (Figures 7C–E).

### 3.5 H_2_S alleviates Dgal-induced mitochondrial dysfunction and oxidative stress

**FIGURE 8:**
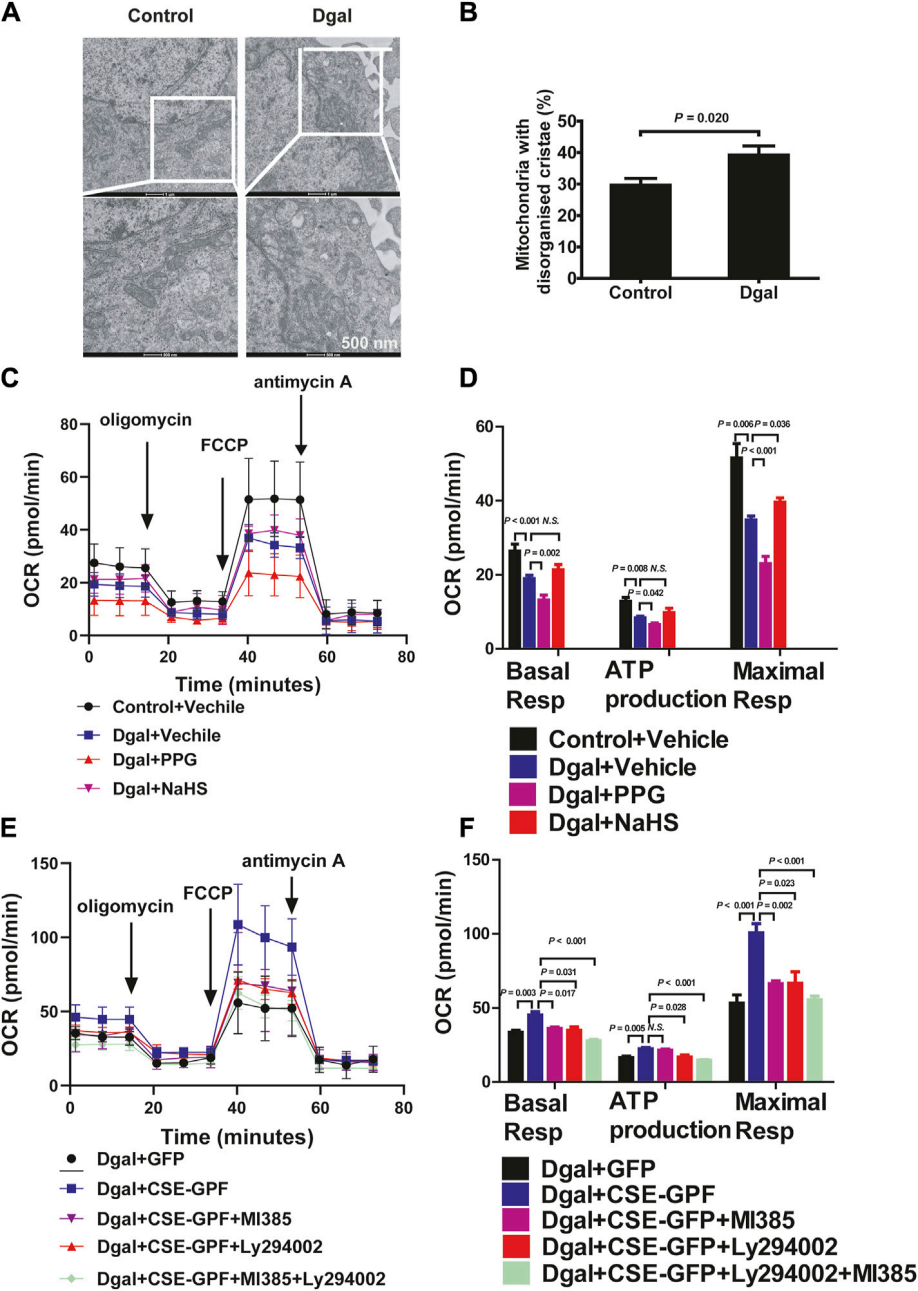
H_2_S significantly increased mitochondrial respiration. **(A, B)** Mitochondria ultrastructure in the HUVECs was examined by transmission electron microscopy (TEM). Representative images and quantitative analysis of the mitochondrial ultrastructure in the control and Dgal-induced accelerated-aging HUVECs. Scale bar = 500 nm (n = 3). **(C, D)** Measure of OCR and corresponding quantitative analysis of exogenous H_2_S treatment on HUVECs (n = 11). **(E, F)** Measure of OCR and corresponding quantitative analysis of endogenous H_2_S treatment on HUVECs (n = 9). FCCP, trifluoromethoxy carbonyl cyanide phenylhydrazone. The values are expressed as means ± SEM, with *p* < 0.05 considered as significant.

We examined the mitochondrial ultrastructure and functions as mitochondrial dysfunction plays a crucial role in oxidative stress and senescence. TEM revealed that Dgal-treated HUVECs exhibited mitochondrial ultrastructural changes, including irregular arrangement and loss of cristae (Figure 8A), with a significantly increased proportion (approximately 40%) of mitochondrial ultrastructure disorders (Figure 8B). The seahorse assay showed that Dgal-treated HUVECs exhibited a significant decrease in mitochondrial respiration (ATP production, basal and maximal respiration). Respiration ability was further decreased in the PPG group, while the exogenous H_2_S only alleviated maximal respiration (Figures 8C,D). Increases in ATP production and basal respiration were observed but were not statistically significant (Figures 8C,D). From the measurements of the endogenous H_2_S and PI3K/AKT effects, CSE overexpression was observed to reverse Dgal-induced mitochondrial dysfunction in the HUVECs (ATP production, basal and maximal respiration), while these were blocked by the PI3K/AKT and NRF2 inhibitors (Figures 8E,F).

**FIGURE 9:**
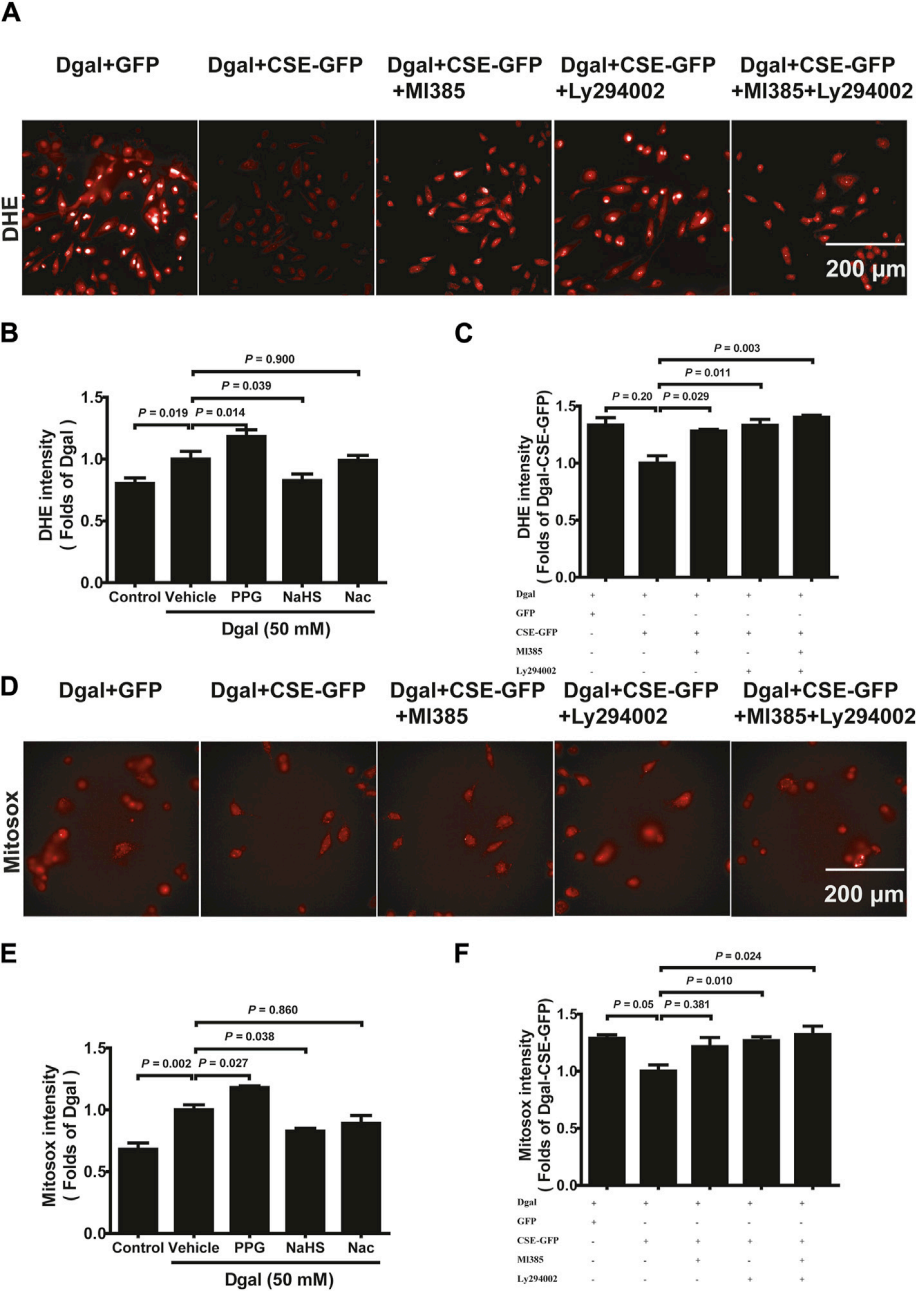
H_2_S decreased endothelial cell reactive oxygen species production mitigated by PI3K/AKT signaling. **(A, B, C)** Representative images and quantitative analysis of the DHE staining samples (n = 8). **(D, E, F)** Representative images and quantitative analysis of the MitoSox staining samples (n = 8). The values are expressed as means ± SEM, with *p* < 0.05 considered as significant.

Mitochondrial dysfunction often leads to increased ROS (Newgard and Sharpless, 2013). DHE intensity was elevated in Dgal-induced accelerated senescence in the HUVECs relative to that in the control (Figures 9A,B, Supplementary Figure S2A). The DHE intensity of the PPG group was higher than that of the Dgal group but significantly decreased in the exogenous H_2_S case (Figures 9A,B). Additionally, the DHE fluorescence intensity levels decreased upon CSE overexpression and were blocked by the PI3K/AKT and NRF2 inhibitors (Figure 9C). Similar protective effects were observed for both exogenous and endogenous H_2_S in mitochondrial oxidative stress based on the MitoSox assay, while the MitoSox fluorescence intensity levels decreased upon CSE overexpression and were only blocked with the PI3K/AKT inhibitor but not the NRF2 inhibitor (Figures 9D–F, Supplementary Figure S2B). These data demonstrate that Dgal-induced oxidative burden is correlated with mitochondrial dysfunction.

### 3.6 H_2_S affects mitochondrial function and NRF2 nuclear translocation

**FIGURE 10:**
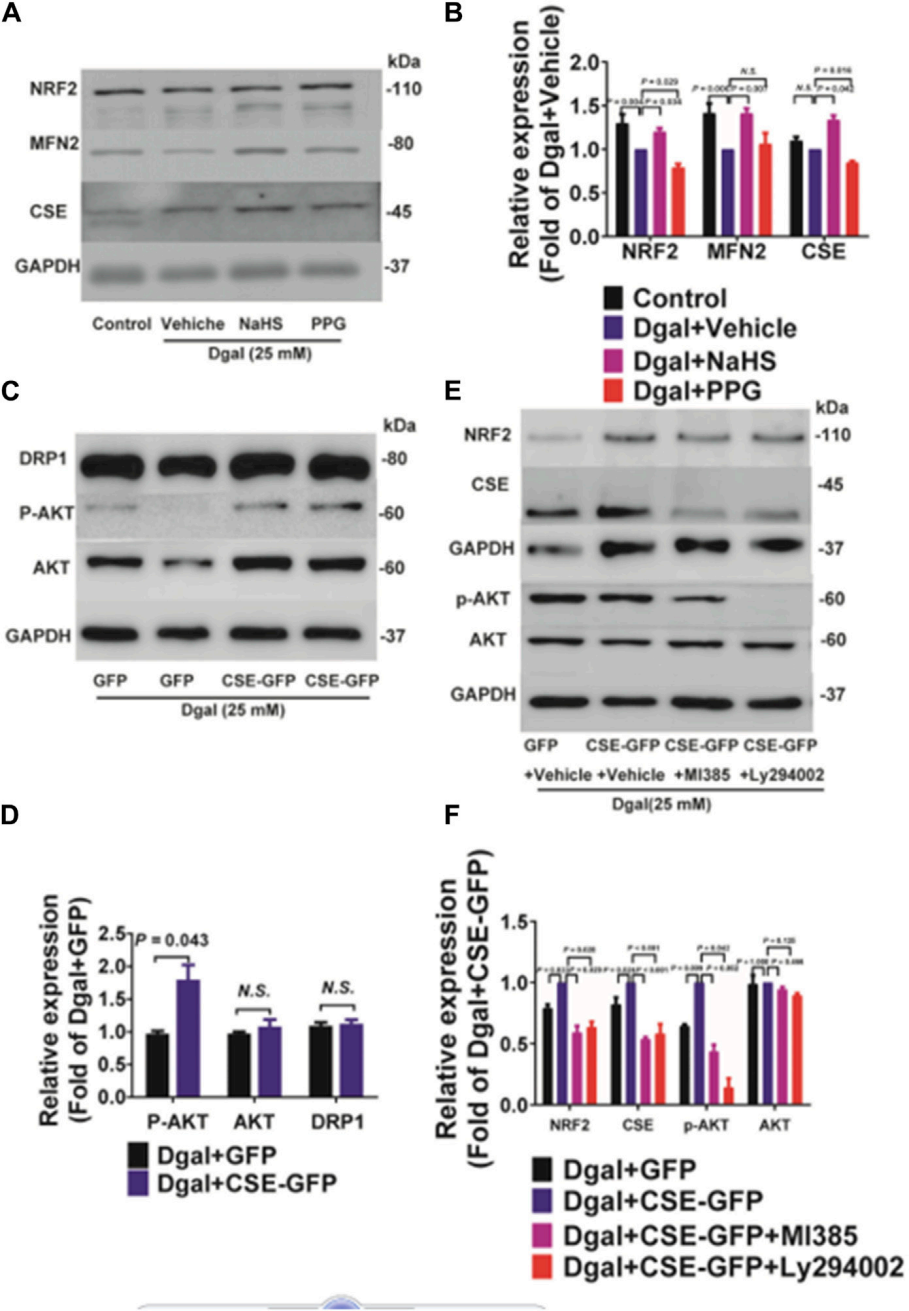
Both exogenous and endogenous H_2_S treatments regulate mitochondria-related proteins and NRF2 in accelerated-aging HUVECs. **(A, B)** Representative immunoblotting images and quantification of the expressions of NRF2 and MFN2 in accelerated-aging HUVECs, following pretreatment with exogenous H_2_S and PPG (n = 6). **(C, D)** Representative immunoblotting images and quantification of the expressions of DRP1, p-AKT, and AKT in accelerated-aging HUVECs, following CSE overexpression (n = 6). **(E, F)** Representative immunoblotting images and quantification of the expressions of NRF2, CSE, p-AKT, and AKT in accelerated-aging HUVECs, following CSE overexpression and pretreatment with NRF2 and/or PI3K/AKT inhibitors (n = 6). The values are expressed as means ± SEM, with *p* < 0.05 considered as significant.

The GO and KEGG analyses showed that the PI3K/AKT signaling pathway and ATPase activity-related signaling regulation were significantly affected by CSE overexpression in the accelerated-aging HUVECs (Figure 3). To determine the effects of CSE on the mitochondria in accelerated senescent HUVECs, we measured the expressions of the mitochondrial fusion regulators mitofusin2 (MFN2) and dynamin-1-like (DRP1), as well as the NRF2 and PI3K/AKT signaling pathways. The CSE protein expression decreased in the accelerated-aging HUVECs (Supplementary Figure S3A). MFN2 and NRF2 expressions decreased after incubation with Dgal for 48 h and were partially reversed upon treatment with exogenous H_2_S (50 μM) (Figures 10A,B). For the endogenous H_2_S, pretreatment with overexpressed CSE resulted in overactivation of PI3K/AKT (Figures 10C,D), which was in line with the RNA sequencing results (Figure 3). Additionally, we found that NRF2 expression increased after infection with CSE and decreased after treatment with the NRF2 and PI3K/AKT inhibitors (Figures 10E,F).

**FIGURE 11:**
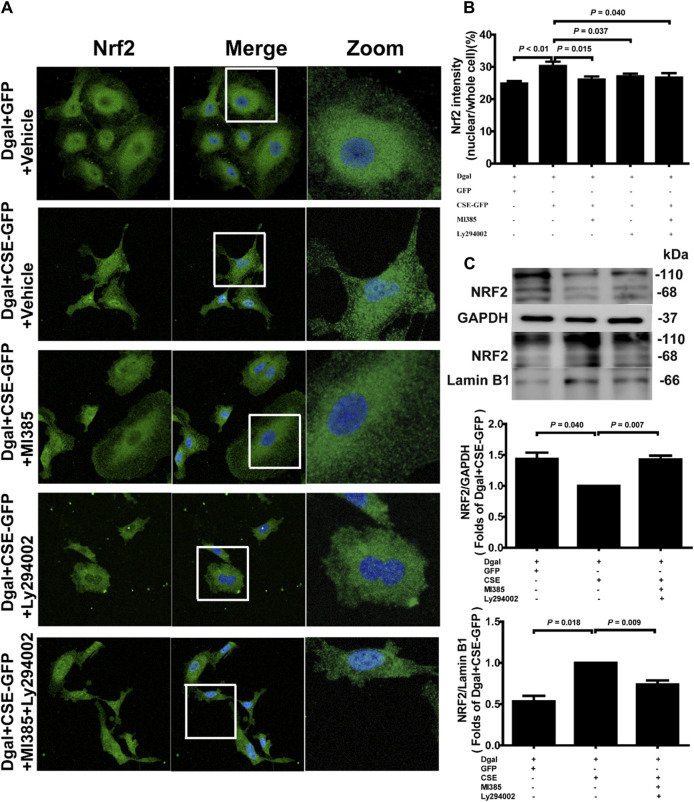
Endogenous H_2_S treatment regulates NRF2 nuclear translocation through PI3K/AKT signaling. **(A, B)** Representative immunofluorescence images and quantification of NRF2 in accelerated-aging HUVECs. Three biological replicates were considered, and each biological replicate was quantified with at least 15 cells. **(C, D, E)** Representative immunoblotting images and quantification of the expression of NRF2 between the cytoplasm and nucleus, following CSE overexpression and pretreatment with NRF2 and PI3K/AKT inhibitors (n = 6). The values are expressed as means ± SEM, with *p* < 0.05 considered as significant.

Our previous study showed that exogenous H_2_S treatment induced NRF2 translocation from the cytoplasm to the nucleus (Hou et al., 2017). We also observed that the percentage of nuclear/whole cells increased after CSE overexpression and decreased upon treatment with the NRF2 and PI3K/AKT inhibitors (Figures 11A,B). Western blotting was performed to confirm the NRF2 translocation tendency (Figures 11C–E).

### 3.7 CSE translocates into the mitochondria upon oxidative stress and is altered by translocase of the outer mitochondrial membrane protein 70 (TOMM70)

**FIGURE 12:**
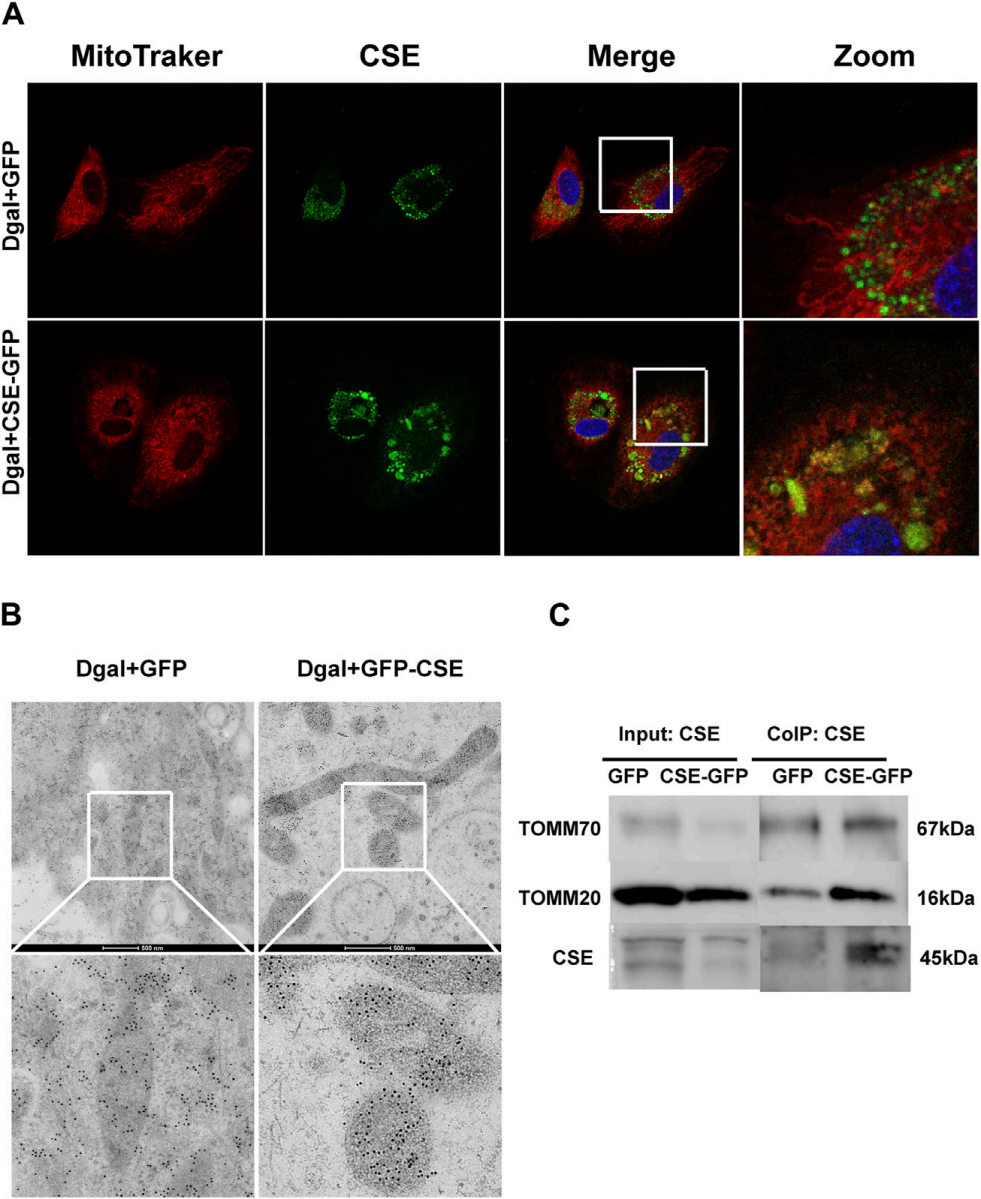
CSE translocation into the mitochondria under stressed conditions. **(A)** Representative immunofluorescence images of CSE in accelerated-aging HUVECs (n = 3). **(B)** Representative immunoelectron microscopy images of CSE in accelerated-aging HUVECs (n = 2). **(C)** Representative co-IP images of TOMM20, TOMM70, and CSE in accelerated-aging HUVECs (n = 3).

**FIGURE 13:**
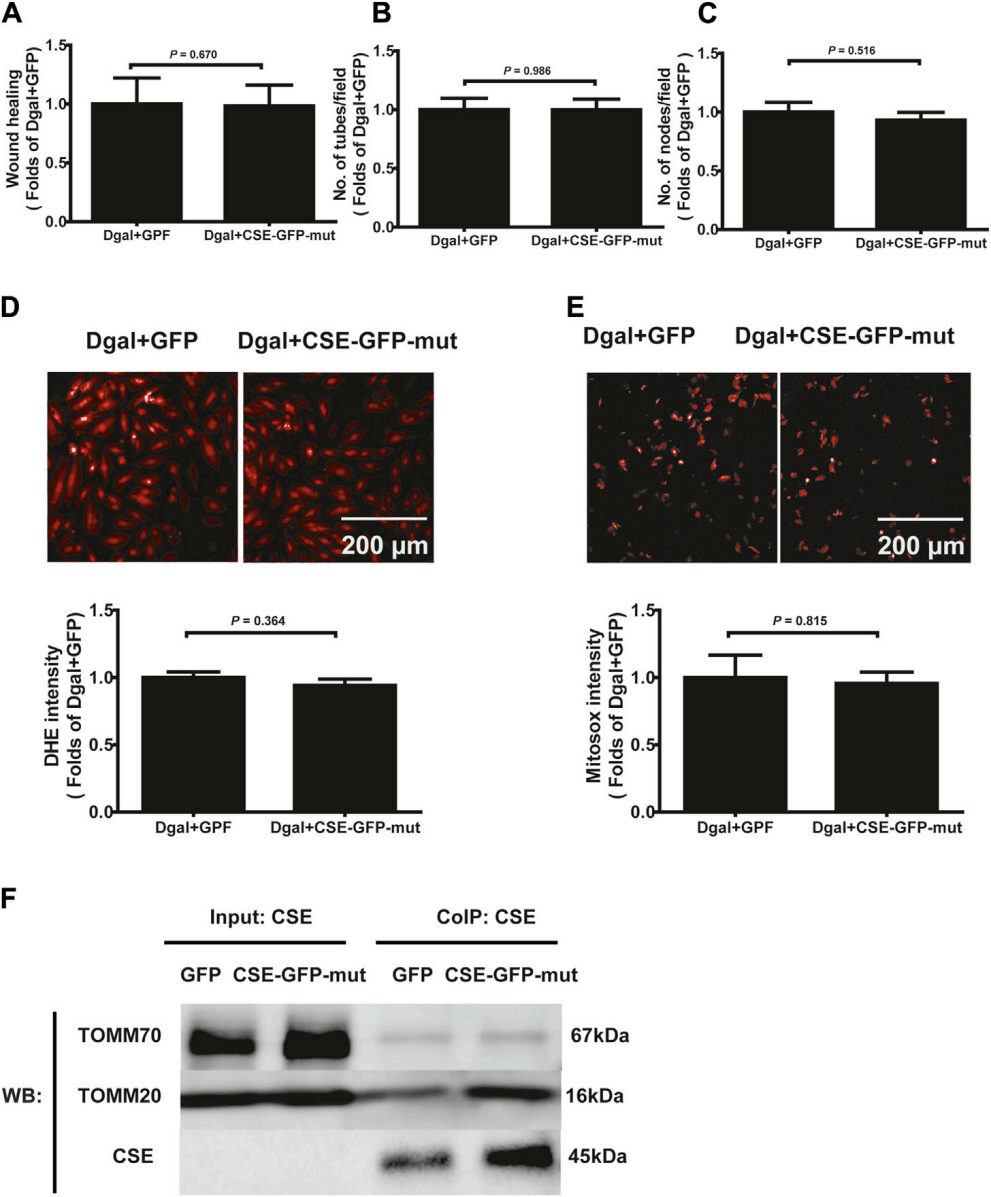
Effects of the mutant CSE protein activity center. **(A, B, C)** Quantification of scratch wound healing, tubes, and branching point number for treatment with CSE-GFP-mut virus (CSE^D187A^) (n = 8). **(D, E)** Representative images and quantitative analysis of DHE and MitoSox staining samples (n = 12). **(F)** Representative co-IP images of TOMM20, TOMM70, and CSE in accelerated-aging HUVECs (n = 3). The values are expressed as means ± SEM, with *p* < 0.05 considered as significant.

Next, we used immunofluorescence colocalization and immunoelectron microscopy to examine the translocation of CSE. As shown in Figure 12A, CSE colocalized with mitochondria (MitoTracker showed mitochondria), and this tendency was much greater in the Dgal + GFP-CSE group than in the control group. We observed more number of black/gold particles (CSE protein locations) in the mitochondria upon CSE overexpression in the accelerated senescent HUVECs (Figure 12B). Next, we overexpressed CSE during accelerated senescence of the HUVECs and performed mass spectrometry measurements; the corresponding results showed that TOMM70 increased significantly upon CSE overexpression, suggesting a close interaction between CSE and TOMM70 (Supplementary Table S2). Immunofluorescence co-immunoprecipitation (co-IP) confirmed that the expressions of TOMM20 and TOMM70 increased following CSE overexpression and that CSE was associated with both TOMM20 and TOMM70 (Figure 12C). To further evaluate the CSE protein function, we constructed a CSE-GFP-mut virus (CSE^D187A^). The ability of CSE to catalyze H_2_S production decreased after mutation of one of the important activity centers (CSE^D187A^) (Supplementary Figure S1E). After pretreatment with CSE-GFP-mut, we found that migration promotion and tube formation had disappeared (Figures 13A–C) along with the antioxidative effect, as confirmed by the DHE and MitoSox assays (Figures 13D,E). The results of co-IP showed that TOMM20 expression increased, whereas TOMM70 expression remained the same following CSE-GFP-mut overexpression (Figure 13F).

## 4 Discussion

In this study, we used an accelerated-aging endothelial cell model to assess the effects of external and internal H_2_S. Our work revealed two important findings. 1) Both exogenous and endogenous H_2_S have protective roles in Dgal-induced accelerated senescence of endothelial cells, and this protective effect partly promotes endothelial angiogenesis and mitochondrial function preservation through the AKT/NRF2 signaling pathway. 2) CSE translocates into the mitochondria to produce H_2_S nearby under stressed conditions and plays an antioxidant role in the accelerated senescent endothelial cells; CSE mutation partially reduces such protective ability to promote angiogenesis, resist oxidative stress, and enter the mitochondria. Therefore, CSE mutation (CSE^D187A^) may be a potential target for drug therapy in the future.

Population aging is a global phenomenon that places high demands on healthcare systems and the society (Zhang et al., 2015). Incubation with low-dose Dgal *in vitro* (1–100 g/L for 48 h) and *in vivo* (125 mg/kg/day for 8 weeks) is a generally accepted model for accelerated aging studies (Yang et al., 2008). Based on the findings of Chen et al. (2019) and our previous study (Hou et al., 2016), Dgal exposure leads to oxidative-stress-induced aging, which is similar to the natural aging process. Therefore, we used a Dgal-induced accelerated aging model to investigate aging and age-related diseases. Emerging evidence has shown that patients with atherosclerosis, hypertension, or diabetes may have lower plasma H_2_S levels (Carmeliet, 2000; Jain et al., 2010; Jain et al., 2012). We previously demonstrated that endogenous H_2_S levels were significantly lower in naturally aging mouse kidney tissue and plasma (Hou et al., 2016) as well as accelerated aging of the mouse heart, liver, and kidney tissues (Wu et al., 2017). In the present study, reduced endogenous H_2_S levels were observed in a Dgal-induced accelerated senescent HUVEC model. Therefore, decreased H_2_S levels may be common during aging. The collection of plasma samples from healthy young and old adults is necessary to verify this conclusion, which is one of the limitations of this study. Angiogenesis is characterized by remodeling of the extracellular matrix and changes in the endothelial cell behaviors, resulting in cell growth, migration, and assembly into capillary structures (Li et al., 2021). Excessive or insufficient growth of new blood vessels can cause angiogenesis-related diseases, such as coronary artery disease, cancer, and diabetes, in addition to aging (Ong and Hausenloy, 2010; Kim and Byzova, 2014). Cai et al. (2007) first reported that H_2_S is angiogenic dependent on AKT phosphorylation; in the present study, we assessed whether H_2_S played a protective role against aging via PI3K/AKT signaling. Based on RNA-seq and Western blotting results, we found that the PI3K/AKT signaling pathway was activated after infection with the CSE adenovirus in Dgal-induced accelerated senescence of HUVECs. The angiogenic abilities of the accelerated-aging endothelial cells decreased, which was partially reversed by both exogenous and endogenous H_2_S, and the protective effects were also partially dependent on PI3K/AKT and NRF2 signaling (Figures 5, 6). Thus, recovery of endogenous H_2_S production may contribute to the promotion of angiogenesis and antiaging processes.

Numerous studies have shown that mitochondria are closely related to endothelial homeostasis and angiogenesis (Elrod et al., 2007; Lugus et al., 2011). However, their underlying molecular mechanisms in endothelial cells remain unclear. Alam et al. (2023) reported that H_2_S attenuates myocardial ischemia-reperfusion injury by preserving mitochondrial function and caspase-3 activation. We observed that Dgal-induced accelerated senescence inhibited mitochondrial respiration in HUVECs, with the cells exhibiting antiapoptotic status; both exogenous and endogenous H_2_S could partially reverse this condition, and the regulation was partially dependent on PI3K/AKT and NRF2 signaling (Figures 7, 8). Dong et al. (2024) showed that ceramide-1-phosphate disrupts the interactions between NRF2 and KEAP1, thus allowing NRF2 nuclear translocation to play an antioxidative role. Olson et al. (2008) reported that NRF2 promotes mitochondrial sulfur metabolism by increasing cellular cysteine availability; mitochondrial activation induced by NRF2 activation is dependent on cystine uptake owing to KEAP1 inhibition (Olson et al., 2008). Thus, NRF2 inhibitors may regulate mitochondrial functions. In this study, we observed that CSE overexpression enhanced mitochondrial functions (ATP production, basal and maximal respiration) and that these effects were reversed by incubation with an NRF2 inhibitor (Figure 8).

Fu et al. (2012) first proved that mitochondria produced H_2_S nearby after CSE translocation. Ellwood et al. (2021) reported that AP39 (100 p.m.), a mitochondria-targeted H_2_S compound, can improve *C. elegans* function in the Duchenne muscular dystrophy (DMD) model and that its protective effects are based on the mitochondria. The symptoms of DMD (characterized by progressive muscle degeneration and weakness due to dystrophin gene mutations) are similar to those associated with accelerated aging. In this study, we found that CSE could translocate into the mitochondria under Dgal-induced accelerated-aging conditions, in line with the findings of a previous study (Fu et al., 2012), and that this translocation was dependent on the CSE protein active center (CSE^D187A^) (Figures 12, 13). Fu et al. (2012) noted that the percentage of CSE translocation into the mitochondria was dependent on the expression of TOMM20 in the smooth muscle cells; they also noted that the binding of TOMM20 could be the prerequisite for CSE mitochondrial translocation (Fu et al., 2012). H_2_S acts as an oxygen sensor by sensing the oxygen levels in the mitochondria and regulating ATP production under different conditions (Olson et al., 2008). We observed that CSE translocation played a role in antioxidative and pro-angiogenesis activities depending on the CSE protein center (Figures 12, 13), which was consistent with the findings of a previous report (Fu et al., 2012). Furthermore, the antioxidative and pro-angiogenic effects disappeared after CSE mutation (CSE^D187A^) (Figure 13), indicating that the CSE protein activity center could play a key role in its translocation as well as regulation of antioxidative and pro-angiogenesis activities in endothelial cells. These results deepen our understanding of endogenous H_2_S provision in the regulation of mitochondrial energy metabolism in the endothelial cells.

In conclusion, we found that both exogenous and endogenous H_2_S played protective roles against Dgal-induced accelerated aging. Both exogenous and endogenous H_2_S partially promoted endothelial cell angiogenesis and improved mitochondrial functions via the AKT/NRF2 signaling pathway. CSE can translocate into the mitochondria to produce H_2_S nearby and play an antioxidant role in Dgal-induced stress conditions. These results suggest that H_2_S therapy, particularly mitochondrial H_2_S production, could be a promising line of focus for antiaging processes.

## 5 Limitations

We previously reported that the expression levels of endogenous H_2_S-producing enzymes affected Dgal-induced accelerated aging in mice (Hou et al., 2016) and that the H_2_S levels were lower in the heart, liver, and kidney tissues of such mice. The present study is a continuation of our previous research and focused on translocation of the H_2_S-producing enzyme CSE into the mitochondria to produce antioxidant stress effects. This study lacked validation results in Dgal-induced accelerated-aging mice.

## Data Availability

The datasets presented in this study can be found in online repositories. The name(s) of the repository/repositories and accession number(s) can be found in the article/Supplementary Material.

## References

[B1] Alam M. M. Kishino A. Sunget al E. Sekine H. Abe T. Murakami S. (2023). Contribution of NRF2 to sulfur metabolism and mitochondrial activity. Redox Biol. 60, 102624. 10.1016/j.redox.2023.102624 36758466 PMC9941419

[B2] Cai W. J. Wang M. J. Mooreet al P. K. Jin H. M. Yao T. Zhu Y. C. (2007). The novel proangiogenic effect of hydrogen sulfide is dependent on Akt phosphorylation. Cardiovasc Res. 76 (1), 29–40. 10.1016/j.cardiores.2007.05.026 17631873

[B3] Carmeliet P. (2000). Mechanisms of angiogenesis and arteriogenesis. Nat. Med. 6 (4), 389–395. 10.1038/74651 10742145

[B4] Chen B. Sun Y. Zhanget al J. Zhu Q. Yang Y. Niu X. (2019). Human embryonic stem cell-derived exosomes promote pressure ulcer healing in aged mice by rejuvenating senescent endothelial cells. Stem Cell Res. Ther. 10 (1), 142. 10.1186/s13287-019-1253-6 31113469 PMC6528288

[B5] Dong W. Li Q. Lu X. Lan J. Qiu Z. Wang X. (2024). Ceramide kinase-mediated C1P metabolism attenuates acute liver injury by inhibiting the interaction between KEAP1 and NRF2. Exp. Mol. Med. 56, 946–958. 10.1038/s12276-024-01203-4 38556546 PMC11059394

[B6] Ellwood R. A. Hewitt J. E. Torregrossaet al R. Philp A. M. Hardee J. P. Hughes S. (2021). Mitochondrial hydrogen sulfide supplementation improves health in the *C. elegans* Duchenne muscular dystrophy model. Proc. Natl. Acad. Sci. U. S. A. 118 (9). 10.1073/pnas.2018342118 PMC7936346 33627403

[B7] Elrod J. W. Calvert J. W. Morrisonet al J. Doeller J. E. Kraus D. W. Tao L. (2007). Hydrogen sulfide attenuates myocardial ischemia-reperfusion injury by preservation of mitochondrial function. Proc. Natl. Acad. Sci. U. S. A. 104 (39), 15560–15565. 10.1073/pnas.0705891104 17878306 PMC2000503

[B8] Fu M. Zhang W. Wuet al L. Yang G. Li H. Wang R. (2012). Hydrogen sulfide (H2S) metabolism in mitochondria and its regulatory role in energy production. Proc. Natl. Acad. Sci. U. S. A. 109 (8), 2943–2948. 10.1073/pnas.1115634109 22323590 PMC3287003

[B9] Hou C. Li W. Liet al Z. Gao J. Chen Z. Zhao X. (2017). Synthetic isoliquiritigenin inhibits human tongue squamous carcinoma cells through its antioxidant mechanism. Oxid. Med. Cell Longev. 2017, 1379430. 10.1155/2017/1379430 28203317 PMC5292127

[B10] Hou C. L. Wang M. J. Sunet al C. Huang Y. Jin S. Mu X. P. (2016). Protective effects of hydrogen sulfide in the ageing kidney. Oxid. Med. Cell Longev. 2016, 7570489. 10.1155/2016/7570489 27882191 PMC5108860

[B11] Jain S. K. Bull R. Rainset al J. L. Bass P. F. Levine S. N. Reddy S. (2010). Low levels of hydrogen sulfide in the blood of diabetes patients and streptozotocin-treated rats causes vascular inflammation? Antioxid. Redox Signal 12 (11), 1333–1337. 10.1089/ars.2009.2956 20092409 PMC2935346

[B12] Jain S. K. Micinski D. Lieblonget al B. J. Stapleton T. (2012). Relationship between hydrogen sulfide levels and HDL-cholesterol, adiponectin, and potassium levels in the blood of healthy subjects. Atherosclerosis 225 (1), 242–245. 10.1016/j.atherosclerosis.2012.08.036 22989474 PMC3557794

[B13] Kim Y. W. Byzova T. V. (2014). Oxidative stress in angiogenesis and vascular disease. Blood 123 (5), 625–631. 10.1182/blood-2013-09-512749 24300855 PMC3907751

[B14] Li J. Zhang Y. Zenget al X. Cheng Y. Tang L. Hong D. (2021). Lycopene ameliorates insulin resistance and increases muscle capillary density in aging via activation of SIRT1. J. Nutr. Biochem. 99, 108862. 10.1016/j.jnutbio.2021.108862 34530111

[B15] Lohakul J. Jeayeng S. Chaiprasongsuket al A. (2021). Mitochondria-targeted hydrogen sulfide delivery molecules protect against UVA-induced photoaging in dermal fibroblasts, and in mouse skin *in vivo* . Antioxidants Redox Signal. 10.1089/ars.2020.8255 34235951

[B16] Lugus J. J. Ngoh G. A. Bachschmidet al M. M. Walsh K. (2011). Mitofusins are required for angiogenic function and modulate different signaling pathways in cultured endothelial cells. J. Mol. Cell Cardiol. 51 (6), 885–893. 10.1016/j.yjmcc.2011.07.023 21839087 PMC3208756

[B17] Newgard C. B. Sharpless N. E. (2013). Coming of age: molecular drivers of aging and therapeutic opportunities. J. Clin. Invest. 123 (3), 946–950. 10.1172/JCI68833 23454756 PMC3582156

[B18] Olson K. R. Healy M. J. Qinet al Z. Skovgaard N. Vulesevic B. Duff D. W. (2008). Hydrogen sulfide as an oxygen sensor in trout gill chemoreceptors. Am. J. Physiol. Regul. Integr. Comp. Physiol. 295 (2), R669–R680. 10.1152/ajpregu.00807.2007 18565835

[B19] Ong S. B. Hausenloy D. J. (2010). Mitochondrial morphology and cardiovascular disease. Cardiovasc Res. 88 (1), 16–29. 10.1093/cvr/cvq237 20631158 PMC2936127

[B20] Shen X. Pattillo C. B. Pardueet al S. Bir S. C. Wang R. Kevil C. G. (2011). Measurement of plasma hydrogen sulfide *in vivo* and *in vitro* . Free Radic. Biol. Med. 50 (9), 1021–1031. 10.1016/j.freeradbiomed.2011.01.025 21276849 PMC4798232

[B21] Szabo C. (2007). Hydrogen sulphide and its therapeutic potential. Nat. Rev. Drug Discov. 6 (11), 917–935. 10.1038/nrd2425 17948022

[B22] Topf U. Suppanz I. Samluket al L. Wrobel L. Böser A. Sakowska P. (2018). Quantitative proteomics identifies redox switches for global translation modulation by mitochondrially produced reactive oxygen species. Nat. Commun. 9 (1), 324. 10.1038/s41467-017-02694-8 29358734 PMC5778013

[B23] Wang R. (2012). Physiological implications of hydrogen sulfide: a whiff exploration that blossomed. Physiol. Rev. 92 (2), 791–896. 10.1152/physrev.00017.2011 22535897

[B24] Wara A. K. Croce K. Fooet al S. Sun X. Icli B. Tesmenitsky Y. (2011). Bone marrow-derived CMPs and GMPs represent highly functional proangiogenic cells: implications for ischemic cardiovascular disease. Blood 118 (24), 6461–6464. 10.1182/blood-2011-06-363457 21828132 PMC3236127

[B25] Wu W. Hou C. L. Muet al X. P. Sun C. Zhu Y. C. Wang M. J. (2017). H_2_S donor NaHS changes the production of endogenous H2S and NO in D-galactose-induced accelerated ageing. Oxid. Med. Cell Longev. 2017, 5707830. 10.1155/2017/5707830 28512525 PMC5420433

[B26] Xu Y. Li Y. Maet al L. Xin G. Wei Z. Zeng Z. (2018). d-galactose induces premature senescence of lens epithelial cells by disturbing autophagy flux and mitochondrial functions. Toxicol. Lett. 289, 99–106. 10.1016/j.toxlet.2018.02.001 29426000

[B27] Yang G. Wu L. Jianget al B. Yang W. Qi J. Cao K. (2008). H2S as a physiologic vasorelaxant: hypertension in mice with deletion of cystathionine gamma-lyase. Science 322 (5901), 587–590. 10.1126/science.1162667 18948540 PMC2749494

[B28] Zhang D. Yan B. Yuet al S. Zhang C. Wang B. Wang Y. (2015). Coenzyme Q10 inhibits the aging of mesenchymal stem cells induced by D-galactose through Akt/mTOR signaling. Oxid. Med. Cell Longev. 2015, 867293. 10.1155/2015/867293 25789082 PMC4348608

