# Supplementary material for: Cystathionine γ-lyase inhibits mitochondrial oxidative stress by releasing H2S nearby through the AKT/NRF2 signaling pathway
Source: Front Pharmacol. 2024 Jul 23;15:1374720. doi: 10.3389/fphar.2024.1374720 (PMC11300353; doi:10.3389/fphar.2024.1374720)
Supplement: Supplementary file 1 [file Presentation1.PPTX]

## Slide 1
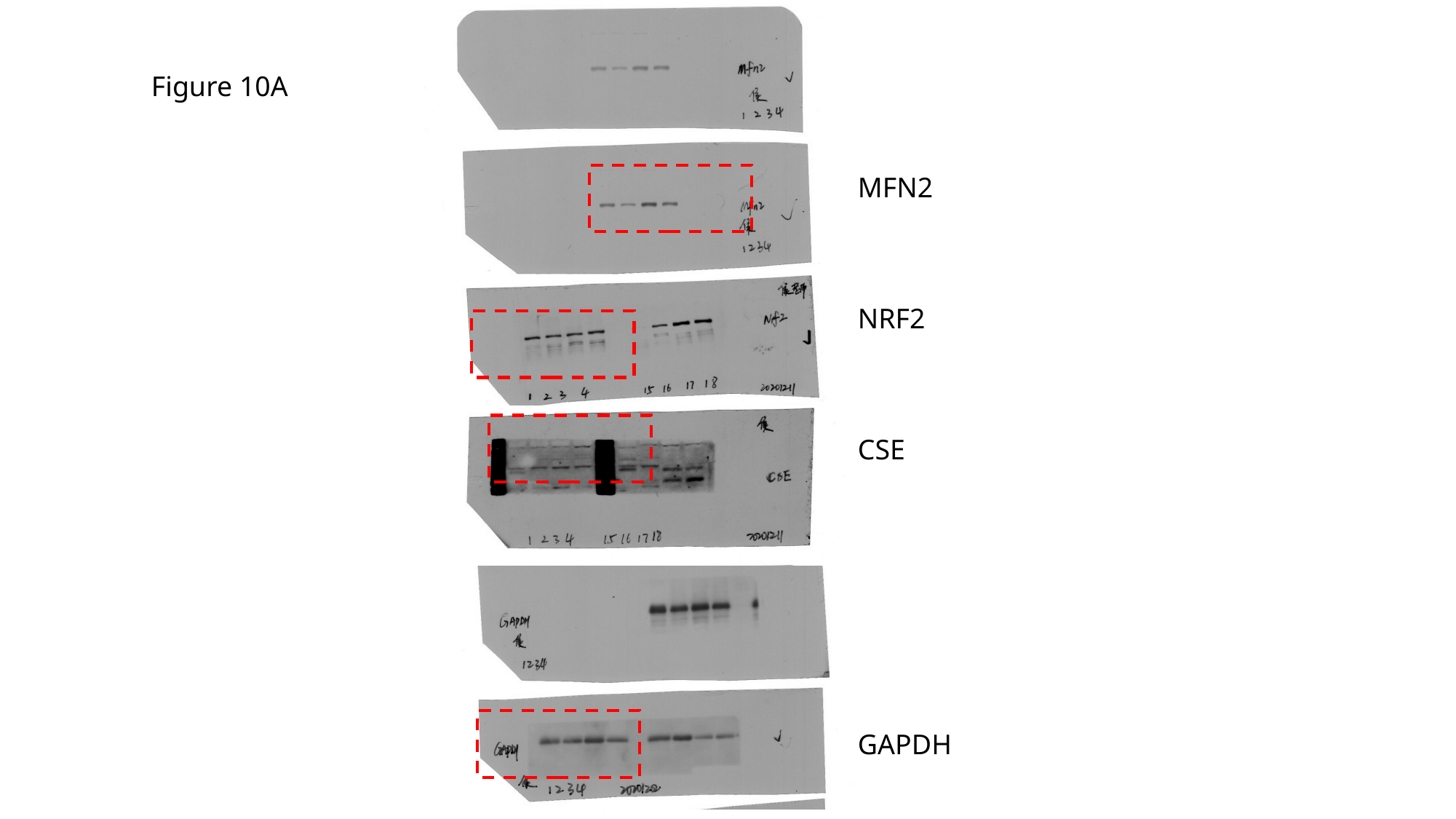

Figure 10A
MFN2
NRF2
CSE
GAPDH

## Slide 2
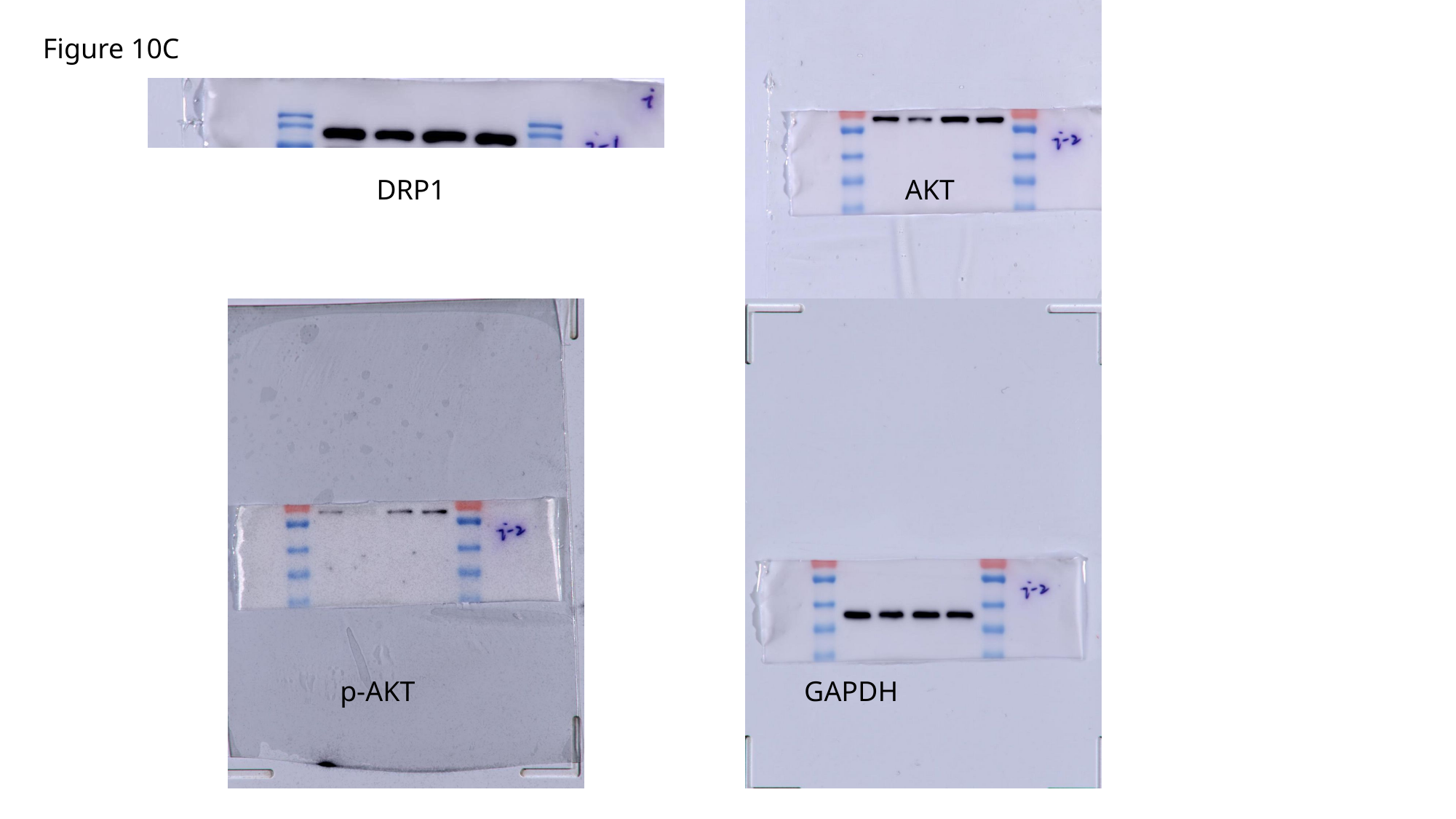

Figure 10C
DRP1 AKT
p-AKT GAPDH

## Slide 3
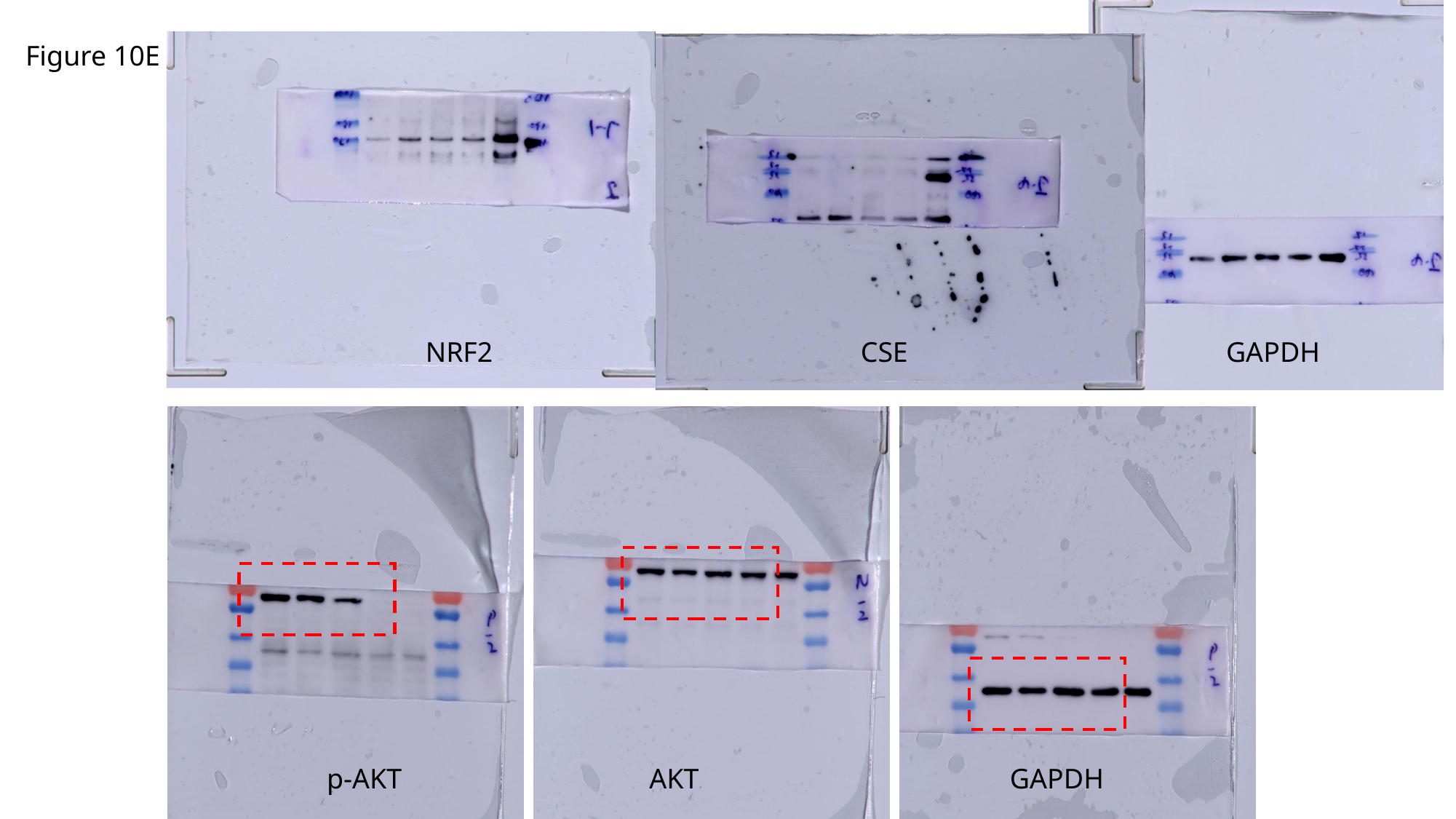

Figure 10E
NRF2 CSE GAPDH
 p-AKT AKT GAPDH

## Slide 4
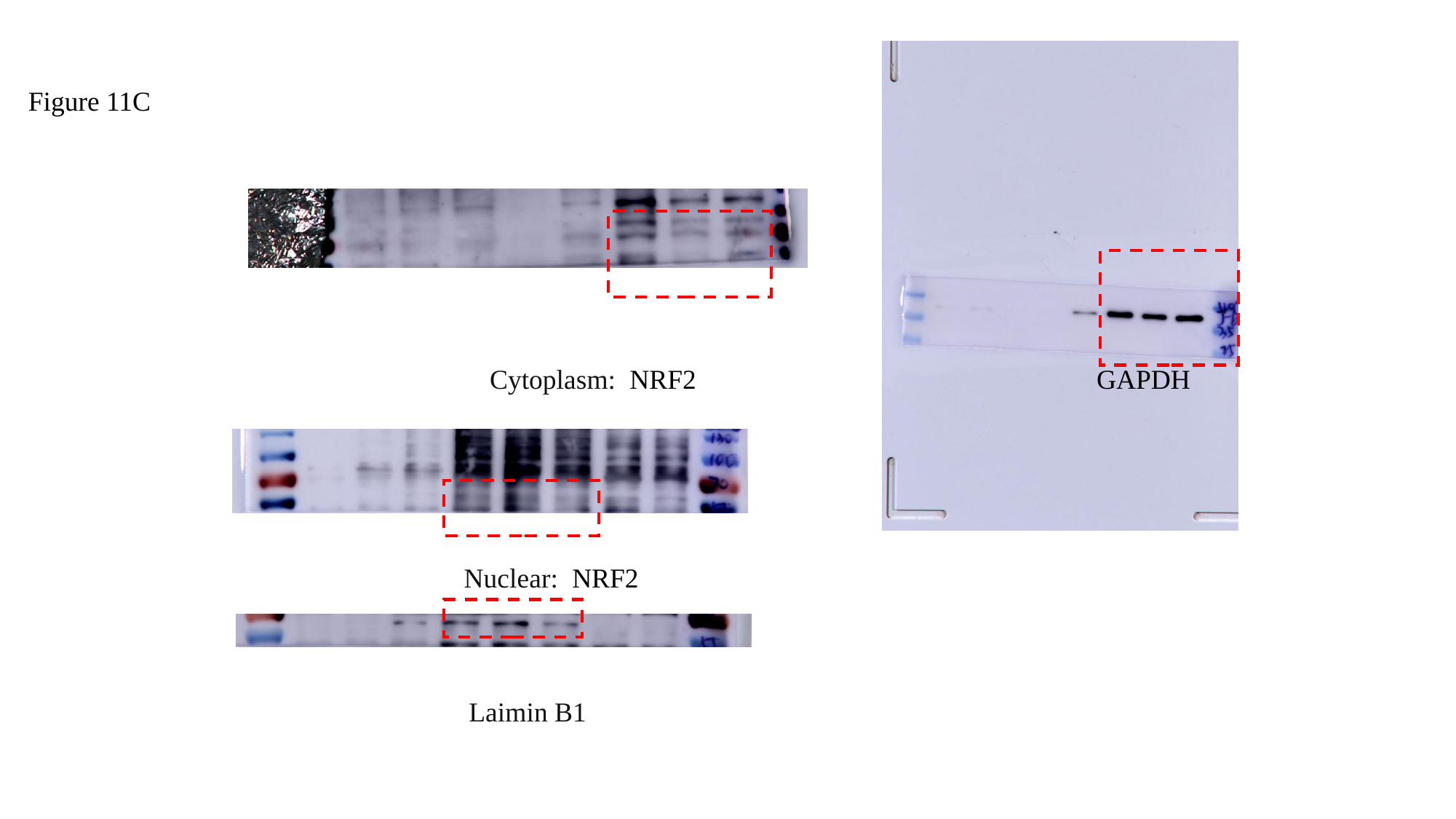

Figure 11C
Cytoplasm: NRF2 GAPDH
Nuclear: NRF2
Laimin B1

## Slide 5
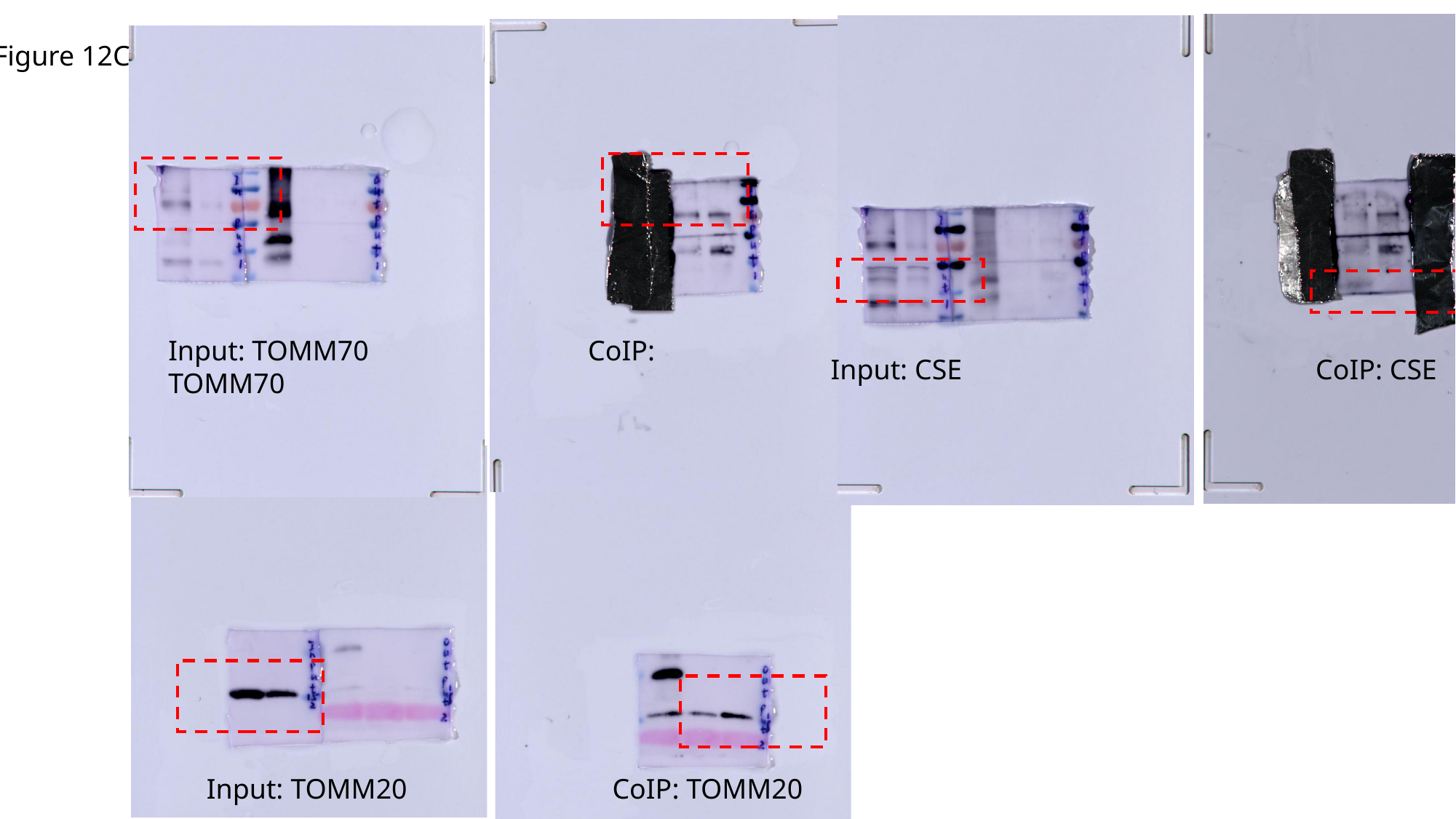

Figure 12C
Input: TOMM70 CoIP: TOMM70
 Input: CSE CoIP: CSE
Input: TOMM20 CoIP: TOMM20

## Slide 6
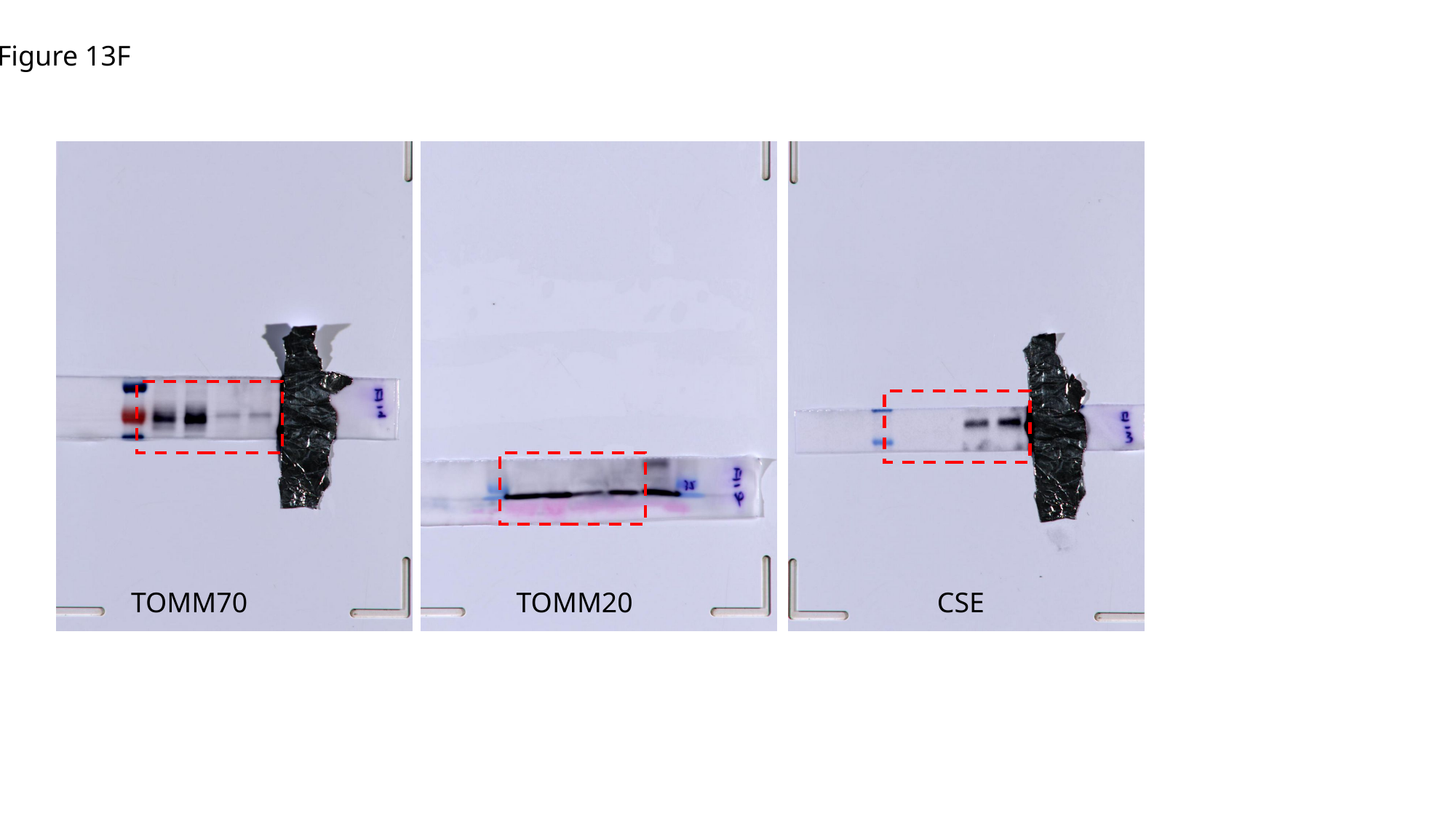

Figure 13F
TOMM70 TOMM20 CSE
