# Supplementary material for: Cystathionine γ-lyase inhibits mitochondrial oxidative stress by releasing H2S nearby through the AKT/NRF2 signaling pathway
Source: Front Pharmacol. 2024 Jul 23;15:1374720. doi: 10.3389/fphar.2024.1374720 (PMC11300353; doi:10.3389/fphar.2024.1374720)
Supplement: Supplementary file 2 [file DataSheet1.docx]

Supporting information


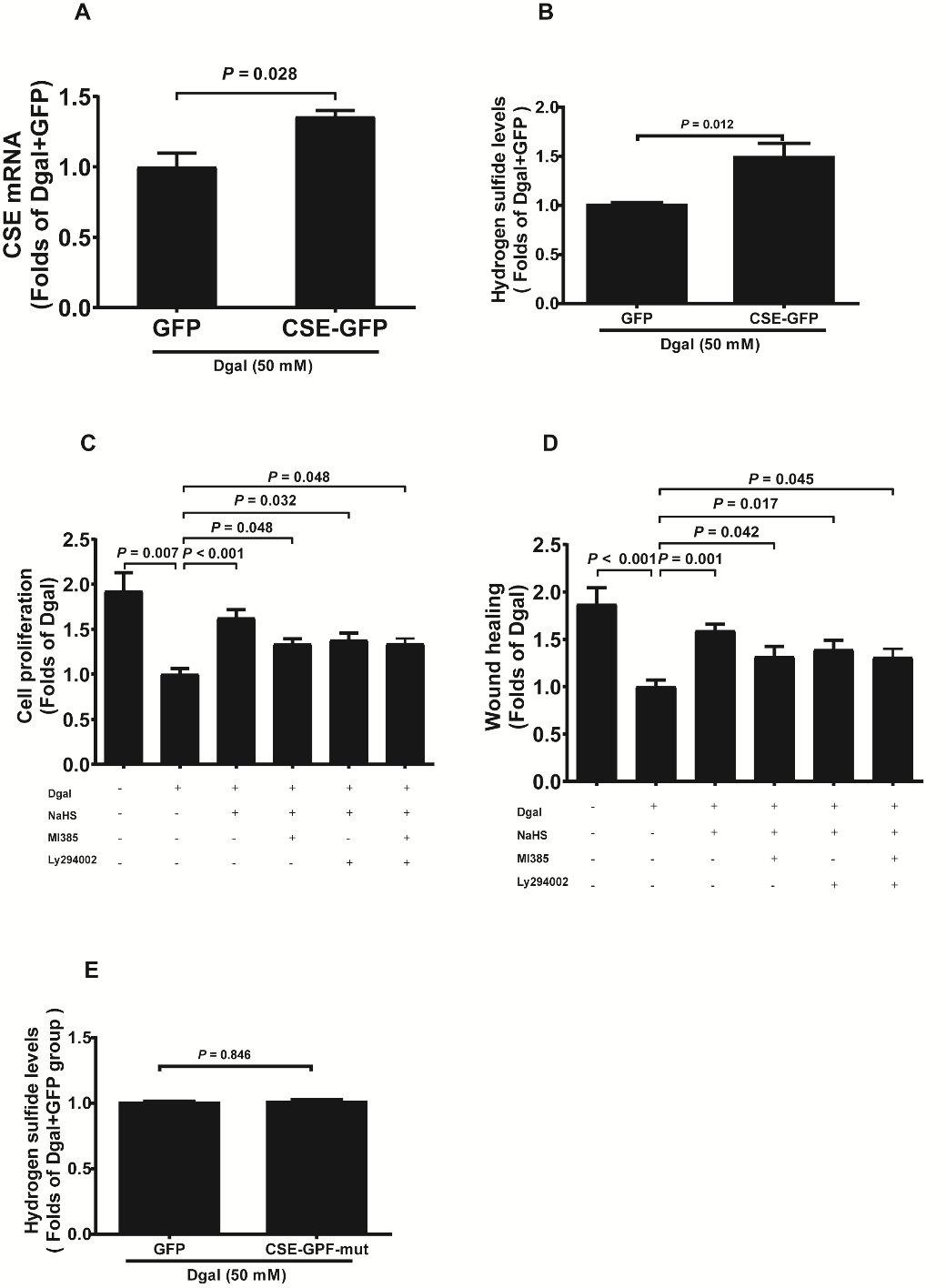


Fig s1

Fig s1. H_2_S levels and CSE mRNA levels were increased significantly after overexpression of CSE in the HUVECs. (A, B) CSE mRNA levels and H_2_S levels in Dgal-induced accelerated aging HUVECs after CSE overexpression (n=8). (C, D) Statistical analysis of the effects of exogenous H_2_S on cell proliferation and migration (n=8). (E) H_2_S levels in Dgal-induced accelerated aging HUVECs after CSE-mut overexpression (n=8). Values are the means ± SE. *P* < 0.05 was considered significant.


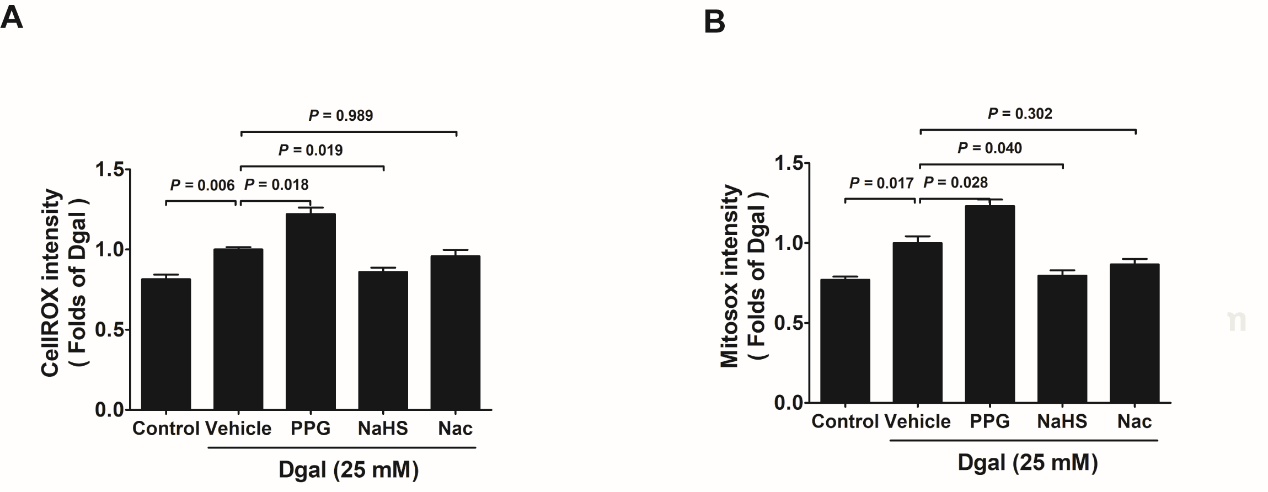


Fig s2. H_2_S decreased endothelial cell reactive oxygen species production. (A, B) Quantitative analysis of the CellROX staining and MitoSox (n=6). Values are the means ± SE. *P* < 0.05 was considered significant.


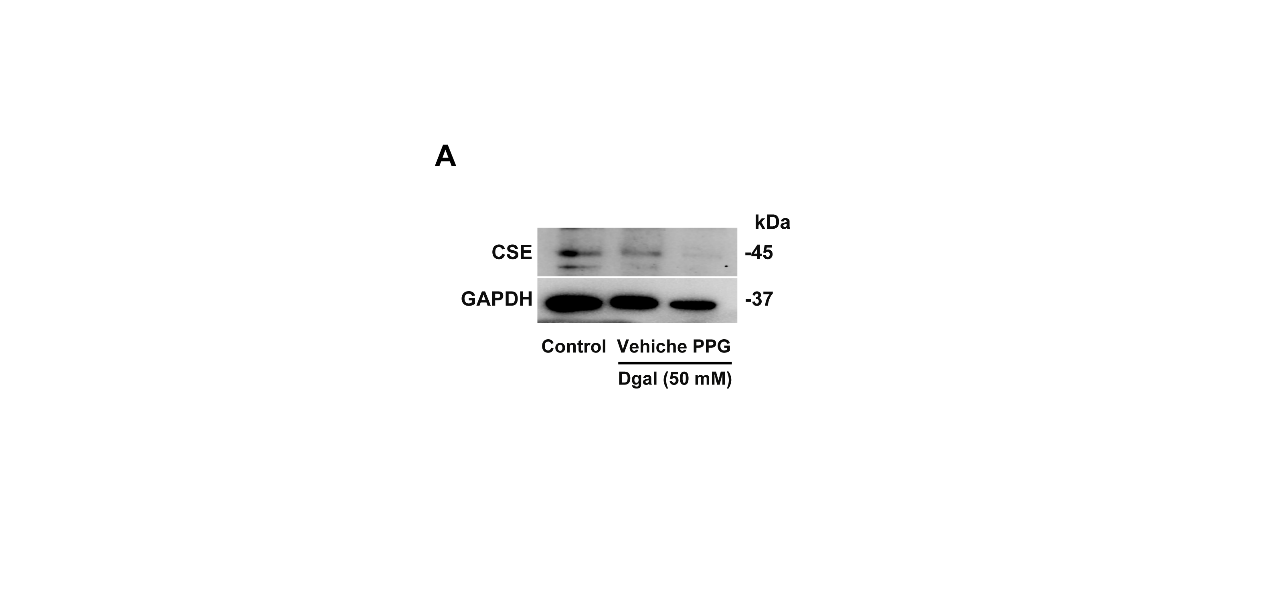


Fig s3

Fig s3. CSE protein expression was decreased in accelerated aging HUVECs. (A) Representative immunoblotting images of the expression of CSE and GAPDH in HUVECs following pretreated with PPG or Dgal (n=3).

Table s1. The relative differently expression genes in Dgal-induced accelerated aging endothelial cells after overexpression CSE through RNA-sequence.

Table s2. All the expression of proteins after CSE overexpression in the Dgal-induced accelerated endothelial cells through mass spectrometry.

Table s1 and Table s2 information was deposited in Figshare.com and can be accessed (https://doi.org/10.6084/m9.figshare.24572191.v1).
